# Single-cell analyses define a continuum of cell state and composition changes in the malignant transformation of polyps to colorectal cancer

**DOI:** 10.1038/s41588-022-01088-x

**Published:** 2022-06-20

**Authors:** Winston R. Becker, Stephanie A. Nevins, Derek C. Chen, Roxanne Chiu, Aaron M. Horning, Tuhin K. Guha, Rozelle Laquindanum, Meredith Mills, Hassan Chaib, Uri Ladabaum, Teri Longacre, Jeanne Shen, Edward D. Esplin, Anshul Kundaje, James M. Ford, Christina Curtis, Michael P. Snyder, William J. Greenleaf

**Affiliations:** 1 Department of Genetics, Stanford University School of Medicine, Stanford, CA USA; 2 Program in Biophysics, Stanford University, Stanford, CA USA; 3 Division of Oncology, Department of Medicine, Stanford University, Stanford, CA USA; 4 Division of Gastroenterology and Hepatology, Department of Medicine, Stanford University, Stanford, CA USA; 5 Department of Pathology, Stanford University, Stanford, CA USA; 6 Department of Computer Science, Stanford University, Stanford, CA USA; 7 Stanford Cancer Institute, Stanford University School of Medicine, Stanford, CA USA; 8 Department of Applied Physics, Stanford University, Stanford, CA USA; 9 Chan Zuckerberg Biohub, San Francisco, CA USA

**Keywords:** Functional genomics, Gene regulation, Epigenetics, Gastrointestinal cancer, Cancer

## Abstract

To chart cell composition and cell state changes that occur during the transformation of healthy colon to precancerous adenomas to colorectal cancer (CRC), we generated single-cell chromatin accessibility profiles and single-cell transcriptomes from 1,000 to 10,000 cells per sample for 48 polyps, 27 normal tissues and 6 CRCs collected from patients with or without germline *APC* mutations. A large fraction of polyp and CRC cells exhibit a stem-like phenotype, and we define a continuum of epigenetic and transcriptional changes occurring in these stem-like cells as they progress from homeostasis to CRC. Advanced polyps contain increasing numbers of stem-like cells, regulatory T cells and a subtype of pre-cancer-associated fibroblasts. In the cancerous state, we observe T cell exhaustion, RUNX1-regulated cancer-associated fibroblasts and increasing accessibility associated with HNF4A motifs in epithelia. DNA methylation changes in sporadic CRC are strongly anti-correlated with accessibility changes along this continuum, further identifying regulatory markers for molecular staging of polyps.

## Abstract (web summary)

Single-cell ATAC-seq and RNA-seq profiling traces the transformation of healthy colon to precancerous adenomas to colorectal cancer (CRC). A large proportion of polyp and CRC cells show a stem-like phenotype.

## Main

The identification of genes and pathways that drive formation of invasive cancers has been the central focus of a number of large-scale genomics efforts^1–3^. These efforts have cataloged the diversity and commonality of many genetic and transcriptional changes that accompany malignancy in diverse cancer types. However, most studies have focused on bulk profiling of advanced stage tumors and have largely ignored premalignant lesions. As a result, a detailed understanding of the progression of phenotypic changes that occur during the transition from normal to precancerous to cancerous state, as well as the molecular drivers of this transformation, remain underexplored.

CRC is an ideal system to study the continuum of phenotypic states along malignant transformation as it follows a stereotyped progression from normal to atypical to carcinoma that includes the formation of precancerous polyps^4,5^, which can subsequently give rise to CRCs. A number of the changes associated with these transitions are nearly universal to all CRC malignancies, as typified by the adenoma-to-carcinoma sequence^6–8^. For example, an estimated 80–90% of colorectal tumors are initiated by loss of APC^9^, resulting in β-catenin stabilization and increased WNT signaling^10^ leading to intestinal hyperplasia^11^. Subsequent mutations in other cancer driver genes such as *KRAS*, *TP53* and *SMAD4* result in the transformation to carcinoma.

Because *APC* mutations are almost universally the initiating event for polyps and CRCs, patients with familial adenomatous polyposis (FAP), who have germline mutations in *APC*, are a suitable population in which to study the natural progression of polyposis. These patients typically develop hundreds of polyps by early adulthood^12,13^, and therefore an individual patient can provide numerous polyps of varied molecular ages and stages of progression, all arising in the same germline background.

To chart the regulatory and transcriptomic changes that occur on the phenotypic continuum from healthy colon to invasive carcinoma, as part of the Human Tumor Atlas Network^14^, we profiled single-nuclei transcriptomes (single-nucleus RNA sequencing (RNA-seq) (snRNA-seq)) and epigenomes (single-cell assay for transposase-accessible chromatin using sequencing (ATAC-seq) (scATAC-seq)) of healthy colon, polyps and CRCs. Many polyps were obtained from patients with FAP who underwent surgical colectomies, allowing both analysis of polyps with diverse sizes and locations of origin, and collection of neighboring unaffected colon tissue. From these single-cell datasets, we first catalog immune, stromal and epithelial cell types. We find large shifts in fibroblast subpopulations that occur along the transition from normal colon to CRC. We identify a subpopulation of exhausted T cells present only in CRC tissue. We observe a much larger fraction of cells exhibiting a stem-like state (both transcriptionally and epigenetically) within polyps and CRCs. We find that polyps populate an epigenetic and transcriptional continuum from normal colon to CRC characterized by sequential opening and closing of chromatin and upregulation and downregulation of genes associated with the cancer state. We identify regulatory elements and transcription factors (TFs) associated with different stages of transformation from normal colon to carcinoma, including early increases in accessibility of regions containing TCF and LEF motifs and loss of accessibility in regions containing KLF motifs. In the final stage of this pathway, malignant transformation, we observe increased accessibility in regions containing HNF4A motifs. Finally, we show that accessibility changes in polyps are strongly anti-correlated with DNA methylation changes in sporadic CRC, and identify a subset of these regions that change their accessibility state early in the malignant continuum, suggesting potential strategies for detection of premalignant polyps.

## Results

### Mapping molecular changes across malignant transformation

**Fig. 1:**
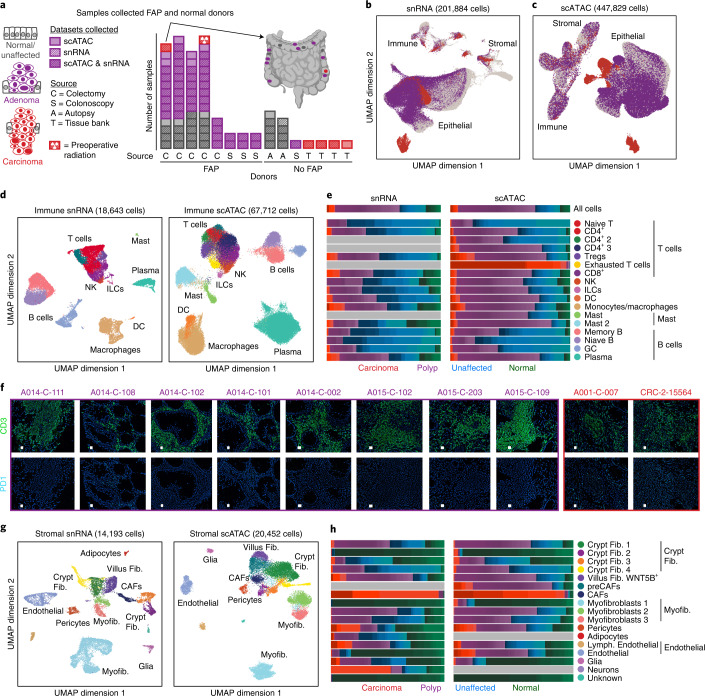
Single-cell atlas of expression and chromatin accessibility in CRC development. **a**, Summary of the samples in this study. The bar chart shows the number of normal/unaffected colon tissues (gray), adenomas (purple) and CRCs (red) assayed for each patient. Locations of samples assayed from a single patient are indicated on the colon on the upper right. These data include deep profiling of four patients with FAP from whom we assayed 8–11 polyps, 0–1 carcinomas and 4–5 matched normal (unaffected) tissues. From non-FAP donors, we collected data on normal colon (9 samples from 2 donors), polyps (1 sample from 1 donor) and CRC tissues (4 samples from 4 patients). **b**,**c**, UMAP representations of all snRNA-seq (**b**) and scATAC-seq (**c**) cells colored by whether the cells were isolated from normal/unaffected colon tissues, adenomas or CRCs. **d**,**g**, UMAP representations and annotations of immune (**d**) and stromal (**g**) cells. **e**,**h**, Fraction of each immune (**e**) and stromal (**h**) cell type isolated from normal (green), unaffected (blue), polyp (purple) and CRC (red) samples. The color gradations within each color represent the contributions of each single sample (for example, each shade of red is a single CRC). **f**, CODEX images of eight polyps and two CRCs where cells are labeled with dark blue, CD3 is labeled in green and PD1 is labeled in light blue. All samples tested are shown in **f**. CODEX imaging of individual specimens was not reproduced. Representative sections of images of the entire specimen are shown in the figure. DC, dendritic cell; Fib., fibroblast; GC, germinal center; ILC, innate lymphoid cell; Myofib., myofibroblast/smooth muscle; NK, natural killer.

**Extended Data Fig. 1:**
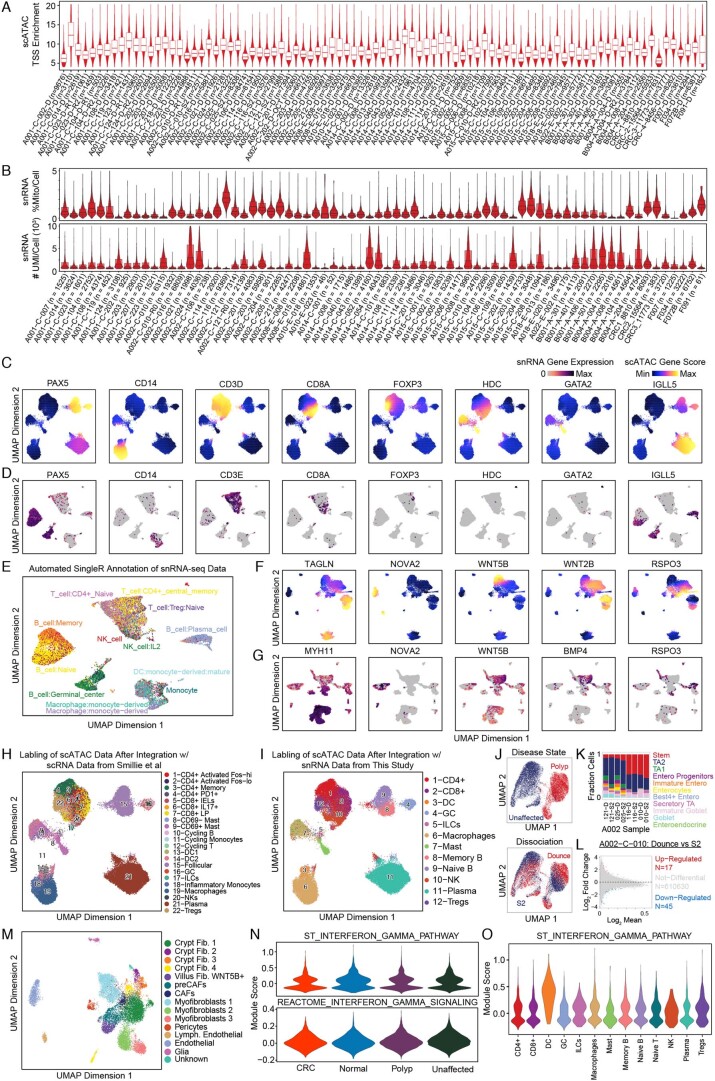
Quality control and annotation of single-cell datasets. **(a)** Violin plots of TSS-enrichments for all scATAC cells from each sample. Samples are labeled by patient (for example A001, A002, etc), source (C = Colectomy, E = Colonoscopy, A = Autopsy, T = Tissue Bank), dissociation (D = dounce, S2 = S2 singulator). Replicates performed on additional sections of the same polyp are indicated with a R. **(b)** Violin plots of the percent of RNA that is mitochondrial RNA per sample and the number of UMIs sequenced for cells from each sample. Samples are labeled the same as in S1A, except all tissues were dounced so the dissociation method is not included. Boxplots represent the median, 25th percentile, and 75th percentile of the data and whiskers represent the highest and lowest values within 1.5 times the interquartile range of the boxplot in A and B. **(c)** UMAP projection of scATAC immune cells colored by gene activity scores reflecting accessibility within and around immune marker genes. **(d)** UMAP projection of snRNA-seq immune cells colored by expression of immune marker genes in each cell. **(e)** UMAP projection of snRNA-seq immune cells colored by automated labeling of snRNA-seq immune cells with SingleR. **(f)** UMAP projection of scATAC stromal cells colored by gene activity scores of stromal marker genes. **(g)** UMAP projection of snRNA-seq stromal cells colored by expression of marker genes. (H, I) UMAP projection of scATAC immune cells where cells are labeled by the nearest snRNA-seq cell from **(h)** Smillie et al or **(i)** this study after integrating the respective datasets with CCA. **(j)** UMAP projections of four scATAC samples with nuclei isolated with both douncing and the S2 Singulator, colored by disease state (top) and dissociation method (bottom). **(k)** Fraction of epithelial cells of each cell type for the 4 samples where nuclei were isolated with douncing and the S2 singulator. **(l)** Differential peaks between scATAC stem cells isolated from two sections of the same polyp that were processed with either the S2 singulator or douncing. **(m)** UMAP representation of stromal cells following Harmony batch correction on LSI dimensions. **(n)** Violin plots of gene module scores for interferon gamma gene sets for immune cells from different disease states. **(o)** Violin plot of gene module scores for an interferon gamma gene set for different immune cell types.

**Extended Data Fig. 2:**
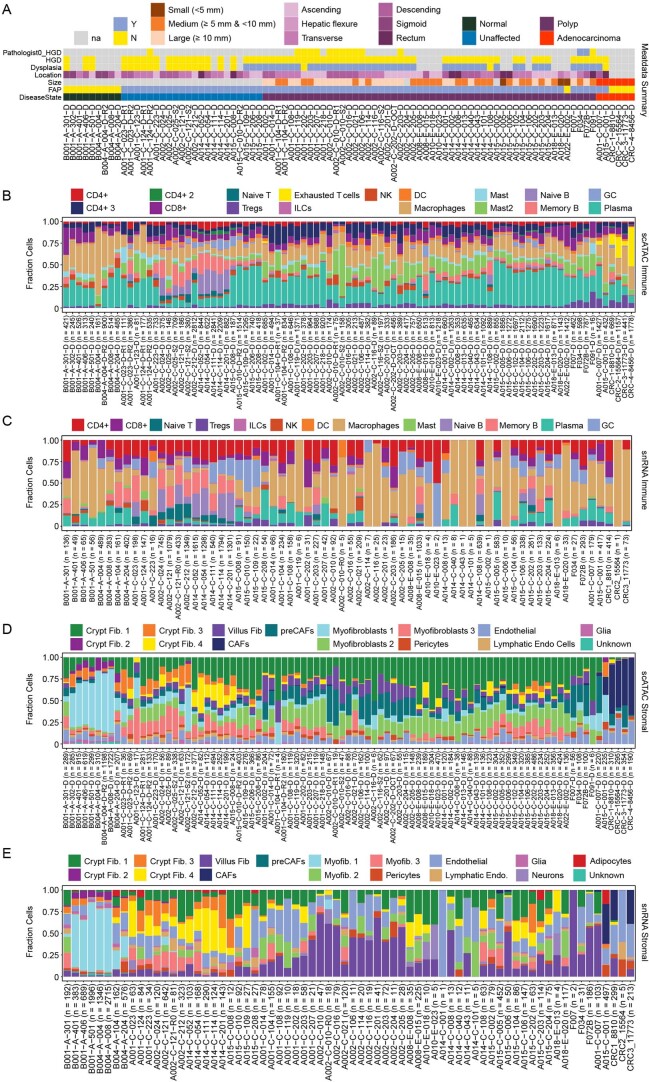
Cellular composition of samples in this study. **(a)** Metadata collected for different samples in this study. (**b, c**) Stacked bar plot representation of the fraction of all immune cells in each sample composed of each cell type for the scATAC (B) and snRNA-seq (**c**) datasets. Each column represents a single sample, with each color representing a different cell type present in the sample. (**d, e**) Stacked bar plot representation of the fraction of all stromal cells in each sample composed of each cell type for the scATAC (**d**) and snRNA-seq (**e**) datasets. Each column represents a single sample, with each color representing a different cell type present in the sample.

We generated single-cell data for 81 samples collected from eight FAP and seven non-FAP donors (Fig. 1a and Supplementary Tables 1 and 2). For each tissue, we performed matched scATAC-seq and snRNA-seq (10x Genomics). We obtained high-quality single-cell chromatin accessibility profiles for 447,829 cells from 80 samples, with a mean transcription start site (TSS) enrichment of ~8 for most samples (Extended Data Fig. 1a). After removing low-quality snRNA-seq cells and samples, we obtained single-cell transcriptomes for 201,884 cells from 70 samples (Extended Data Fig. 1b). Whenever there was sufficient tissue, we generated microscopic pathology data (Extended Data Fig. 2a and Supplementary Table 2) and found the majority of polyps were tubular adenomas, the most common polyp type identified in colonoscopies.

When all snRNA-seq cells (Fig. 1b) and scATAC-seq cells (Fig. 1c) are projected into low-dimensional subspaces, stromal and immune cells generally cluster by cell type whereas epithelial cells largely separate into distinct clusters comprising cells derived from polyps, unaffected tissues or CRCs. As a result, we annotated immune and stromal cells by subclustering cells from all samples, and analyzed epithelial cells separately.

### T cells and myeloid cells are enriched in polyps and CRC

**Extended Data Fig. 3:**
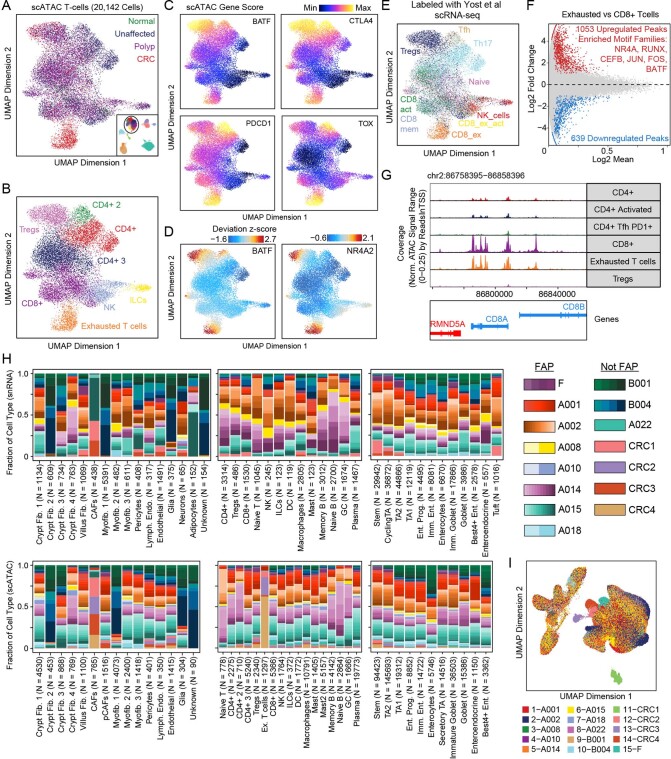
T-cell annotation and donor contributions to clusters of cells. (**a, b**) UMAP projection of all T-cells identified in the scATAC data. Points on the UMAP represent single-cells and are colored by tissue of origin (**c**) and cell type annotations (**d**). **(c**) UMAP projection of scATAC T-cells with cells colored by gene activity scores depicting chromatin accessibility surrounding *BATF*, *CTLA4*, *PDCD1*, and *TOX*. (**d**) ChromVAR deviation z-scores depicting TF motif activity of BATF and NR4A2 plotted on scATAC T-cell UMAP. (**e**) UMAP projection of scATAC T-cells colored by labeling of scATAC-seq T-cells with nearest snRNA-seq T-cells in BCC after integrating the datasets with CCA. (F) MA plot showing differential peaks (Wilcoxon test) between exhausted T-cells and CD8 + T-cells. Motifs with hypergeometric enrichment in peaks more accessible in exhausted T-cells are listed in the plot. (**g**) Genomic tracks depicting accessibility around CD8 locus in T-cell subtypes. Genes on the + strand are indicated in red and genes on the - strand are indicated in blue. (**h**) Stacked bar graph representation of the fraction of each cell type derived from each patient in the study. Cells from each patient have a different color and different shades of the same color represent individual samples from a given patient. (**i**) UAMP representation of all scATAC cells colored by patient of origin.

The immune compartment comprised B cells, T cells, monocytes, macrophages, dendritic cells and mast cells (Fig. 1d). We examined expression of known marker genes (Extended Data Fig. 1c) to annotate snRNA-seq data, and examined chromatin activity scores—a measure of accessibility within and around a given gene body—associated with marker genes to annotate the scATAC cells (Extended Data Fig. 1d). We identified a cluster of exhausted T cells in the scATAC data that exhibited high gene scores of T cell exhaustion marker genes and accessibility at exhausted T cell motifs, and was labeled as exhausted T cells by a published dataset (Extended Data Fig. 3a–g and Methods)^15^.

**Extended Data Fig. 4:**
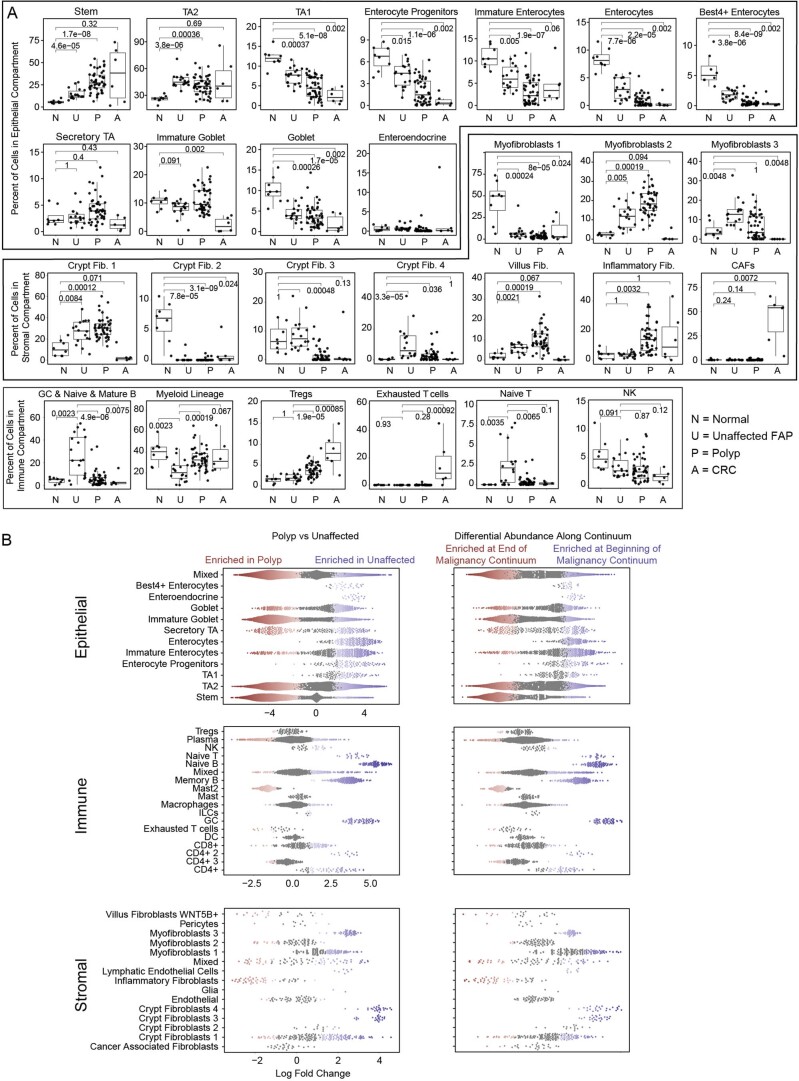
Differential cell type abundance. (**a**) Boxplots depicting the fraction of cells in a given compartment (immune, stromal, or epithelial) that are composed of a given cell type. Each box represents data for a single disease state (N: Normal, U: Unaffected, P: Polyp, A: CRC) in this study. Wilcoxon p-values are listed above the plot and were corrected with Bonferroni correction for multiple hypothesis testing within each cell type. Wilcoxon comparisons were made to normal colon for stromal and epithelial cell types and unaffected FAP colon for immune cell types. The boxplots and statistics are derived from 8 normal samples, 18 unaffected samples, 48 polyp samples, and 6 CRC samples in the epithelial compartment, 8 normal samples, 18 unaffected samples, 47 polyp samples, and 6 CRC samples in the immune compartment, and 8 normal samples, 16 unaffected samples, 46 polyp samples, and 6 CRC samples in the stromal compartment. Boxplots represent the median, 25th percentile, and 75th percentile of the data, whiskers represent the highest and lowest values within 1.5 times the interquartile range of the boxplot, and all points are plotted. (**b**) Milo analysis of differential abundance changes between polyp and unaffected samples. The left plots show comparisons of polyp and unaffected samples, which were selected since they have the greatest number of samples. Neighborhoods that are significantly differentially abundant in polyps are colored in red and neighborhoods that are differentially abundant in unaffected samples are colored in blue. The plots on the right show comparisons along the malignancy continuum, with neighborhoods that are significantly differentially abundant early in the continuum shown in blue and neighborhoods that are significantly differentially abundant late in the continuum shown in red. All comparisons in A and B use the scATAC data, as we had a greater number of scATAC cells.

The cell types identified were present in nearly all samples, although some cell types were enriched or depleted in specific disease states (Fig. 1e and Extended Data Figs. 2b,c, 3h and 4). Significant differences in cell-type abundance were identified with both Wilcoxon testing and a generalized linear model-based method called Milo^16^, which produced consistent results. For example, regulatory T cells (Tregs) were enriched in polyps relative to unaffected tissue, while naive B, memory B and germinal center cells were enriched in unaffected tissues relative to polyps (Extended Data Fig. 4a,b). Enrichment of myeloid cells and specific types of T cells and depletion of B cells was recently reported in a group of 22 mismatch repair-proficient and 13 mismatch repair-deficient CRCs^17^, and we observe similar shifts in the tumor immune composition in precancerous polyps.

The enrichment of (1) Tregs in both polyps and CRC and (2) exhausted T cells in CRC suggests mechanisms of immune evasion in the precancerous and cancerous states^18^. T cell exhaustion, which occurs in response to chronic antigen stimulation and is characterized by reduced cytokine production and increased expression of inhibitory receptors, is thought to be a primary mechanism of immune evasion by cancers^19,20^. To further support the observation of T cell exhaustion only occurring in CRC, we performed CODEX imaging of CD3 and PD1 and found low or undetectable PD1 expression in eight polyps but found PD1 expression in both CRC samples tested (Fig. 1f).

**Extended Data Fig. 5:**
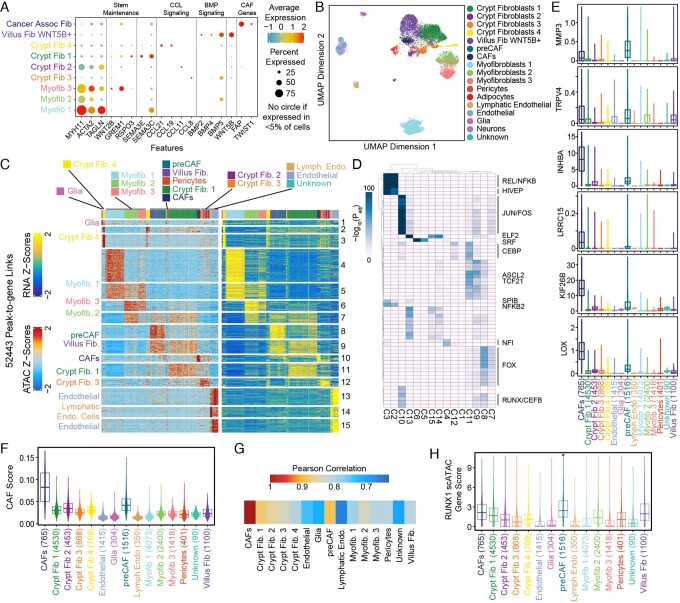
Cell type specific expression and RNA-ATAC integration of stromal cells. (**a**) Dotplot representation of RNA expression of myofibroblast, stem maintenance, SEMA, CCL, BMP, and CAF genes by cells in different fibroblast subtypes. (**b**) Labeling of scATAC cells by aligning scATAC and snRNA-seq data with CCA and labeling scATAC cells based on nearest snRNA-seq cells. (**c**) Peak-to-gene linkages between scATAC and snRNA-seq stromal cells (correlation≥0.45). Rows in the left heatmap represent peaks and are colored by accessibility while rows in the right heatmap represent genes and are colored by expression. (**d**) Hypergeometric enrichment of motifs in clusters of peaks from S5C. (E) Integrated gene expression of CAF marker genes for different stromal cell types. (**f**) CAF scores for different stromal cell types depicted as violin plots with overlying boxplots. CAF scores are a measure of global accessibility at CAF marker peaks, and were defined by first identifying CAF marker peaks relative to all other cell types and then computing the number of Tn5 insertions in those marker peaks for each stromal cell and normalizing by the number of fragments in each cell. (**g**) Pearson correlation between accessibility at all peaks between CAFs and all other stromal cell types. (**h**) Violin plots showing the distribution of RUNX1 scATAC gene scores for cells of each stromal cell type. Boxplots in (**e**), (**f**), and (**h)** represent the median, 25th percentile, and 75th percentile of the data, and whiskers represent the highest and lowest values within 1.5 times the interquartile range of the boxplot.

Within the stromal compartment, we identified glial cells, adipose cells and multiple types of endothelial cells and fibroblasts (Fig. 1g). Fibroblast subtypes include crypt fibroblasts (*WNT2B* or *RSPO3* high), villus fibroblasts (*WNT5B* high) and myofibroblasts (*ACTA2* and *TAGLN* high) (Extended Data Figs. 1f,g and 5a)^21,22^. Consistent with previous results, we observe high expression of BMP signaling genes in villus fibroblasts (Extended Data Fig. 5a). In agreement with recent reports that crypt fibroblasts secrete semaphorins to support epithelial growth, we observe one fibroblast cluster with high expression of semaphorins (Extended Data Fig. 5a)^23^. This cluster of fibroblasts exhibited the highest expression of *RSPO3*, a factor that supports the intestinal stem cell niche^24^. We also observe a cluster of cancer-associated fibroblasts (CAFs) consisting almost exclusively of cells from CRCs, and a scATAC cluster of fibroblasts enriched for cells from polyps and CRCs with accessibility around some of the same genes as CAFs, which we term pre-cancer-associated fibroblasts (preCAFs) (Fig. 1h and Extended Data Figs. 2d,e and 4). These observations suggest that phenotypically distinct fibroblasts exist in polyps and tumors, and thus may play a role in tumorigenesis in precancerous lesions.

We next integrated our scATAC-seq and snRNA-seq datasets to enable analyses of regulatory elements and TFs potentially driving gene expression. We aligned the datasets with canonical correlation analysis (CCA) and assigned RNA-seq profiles to each scATAC-seq cell (integrated expression)^25^. We then labeled scATAC cells with the nearest snRNA-seq cells, which closely agreed with manual immune (Extended Data Fig. 1i) and stromal (Extended Data Fig. 5b) annotations. Finally, we identified peaks highly correlated to gene expression of proximal genes in our datasets, which resulted in 52,443 stromal peak-to-gene links (Extended Data Fig. 5c,d).

### scATAC reveals preCAF population

**Fig. 2:**
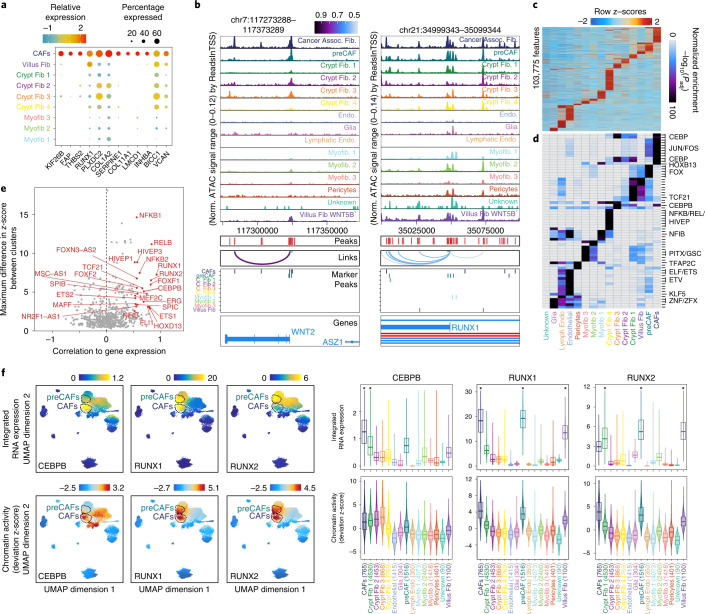
Epigenetic regulators of preCAFs and CAFs. **a**, Dot plot representation of significant (MAST test) marker genes for CAFs. **b**, Genomic tracks for accessibility around *WNT2* and *RUNX1* for different stromal cell types. Peaks called in the scATAC data and peaks-to-gene links are indicated below the tracks. For example, a regulatory element ~50 kb away from the *WNT2* TSS that is most accessible in CAFs whose accessibility is highly correlated to gene expression of *WNT2* is indicated below the tracks. Marker peaks (Wilcoxon FDR ≤ 0.1 and log_2_FC ≥ 1.0) for each fibroblast subtype are indicated below the tracks. **c**, Marker peaks (Wilcoxon FDR ≤ 0.1 and log_2_FC ≥ 0.5) for each stromal cell type. Significance is determined by comparing each cell type with a background of all other cell types. **d**, Hypergeometric enrichment of TF motifs in stromal cell marker peaks. **e**, Plot of maximum difference between chromVAR deviation z-score, depicting TF motif activity, against correlation of chromVAR deviation and corresponding TF expression. TFs with maximum differences in chromVAR deviation z-score in the top quartile of all TFs and a correlation of greater than 0.5 are indicated in red. **f**, RNA expression (top) and chromVAR deviation z-scores (bottom) for selected TFs. The RNA expression plotted is the expression in the nearest RNA cell following integration of the snRNA-seq and scATAC-seq data. Corresponding violin plots and boxplots quantifying integrated gene expression and chromVar deviation z-scores for cells in each cell type are shown at the right. Boxplots represent the median, 25th percentile and 75th percentile of the data, and whiskers represent the highest and lowest values within 1.5 times the interquartile range of the boxplot. Cell types with significantly higher (Wilcoxon test, FDR ≤ 0.01 and log_2_FC ≥ 1) integrated RNA expression when compared with all other cell types are indicated with an asterisk. Assoc., associated; C. Fib, crypt fibroblast; Endo., endothelial; Norm., normalized.

CAFs promote cancer development and progression through diverse mechanisms including matrix remodeling, signaling interactions with cancer cells and perturbation of immune surveillance^26–28^. We observe a CAF cluster with high expression of known CAF marker genes *FAP* and *TWIST1* (Extended Data Fig. 5a)^29,30^. Among the most significant snRNA-seq markers for CAFs were *FAP*, *VCAN* and *COL1A2*, which are involved in extracellular matrix remodeling and upregulated in multiple cancers^30–32^ (Fig. 2a). Specific expression of these genes by CAFs suggests fibroblasts participate in unique extracellular matrix remodeling in cancerous tissues that does not occur in normal colon or precancerous polyps.

While CAFs are known to promote CRC progression, we next explored the role of fibroblasts in precancerous lesions. Because the preCAF cluster was enriched for cells from polyps, we examined accessibility around marker genes for CAFs and found many of these genes more accessible in preCAFs than other fibroblast subtypes. For example, CAFs secrete WNT2 to promote cell proliferation and angiogenesis in CRC^33,34^. CAFs and preCAFs exhibit the greatest accessibility at the *WNT2* TSS (Fig. 2b), suggesting that chromatin changes promote expression of *WNT2* in CAFs and preCAFs. We also observed that preCAFs demonstrated higher integrated expression of multiple CAF marker genes than other fibroblast subtypes (Extended Data Fig. 5e). We computed global CAF accessibility scores for all fibroblast subtypes (Methods) and found that preCAFs had the highest median CAF scores other than CAFs (Extended Data Fig. 5f). Further, accessibility in CAFs was most correlated with preCAFs; however, the correlation with one crypt fibroblast subtype was only slightly lower (Extended Data Fig. 5g). Together, this highlights the similarities between CAFs and preCAFs and suggests that preCAFs may perform similar functions to CAFs.

### RUNX1 is associated with widespread accessibility in CAFs

We found that CAF marker peaks were enriched for JUN/FOS and CEBP motifs and preCAF marker peaks were enriched for JUN/FOS and FOX motifs (Fig. 2c,d and Methods). To nominate TFs driving changes in chromatin accessibility in different stromal cell types, we identified TFs with the highest correlation between their gene expression and the chromatin accessibility activity level of its DNA motif (Fig. 2e, *x* axis). Amongst the most correlated TFs were RUNX1, RUNX2 and CEBPB. We next plotted the expression and motif activities of these TFs on the Uniform Manifold Approximation and Projection (UMAP) representation of the stromal cells and in violin plots grouped by each cell type (Fig. 2f), and noted that chromatin activity levels for RUNX1 and RUNX2, which have similar motifs, are highest in CAFs and preCAFs. However, *RUNX1* is primarily expressed in CAFs and preCAFs, while *RUNX2* has much lower expression in CAFs, suggesting that RUNX1 is a stronger driver of accessibility at RUNX motifs than is RUNX2 in CAFs.

Consistent with the expression of these genes, we observed the greatest accessibility around the *RUNX1* TSS in CAFs and preCAFs (Fig. 2b). When comparing gene scores for each stromal cell type with all other stromal cells, preCAFs had significantly higher *RUNX1* gene scores (log_2_ fold-change (log_2_FC) > 1 and false discovery rate (FDR) < 0.01), and no other cell types met this significance threshold. When identifying accessibility closest to *RUNX1*, we found five significant marker peaks for preCAFs and four for CAFs (Fig. 2b).

### Polyps are enriched for stem-like epithelial cells

**Fig. 3:**
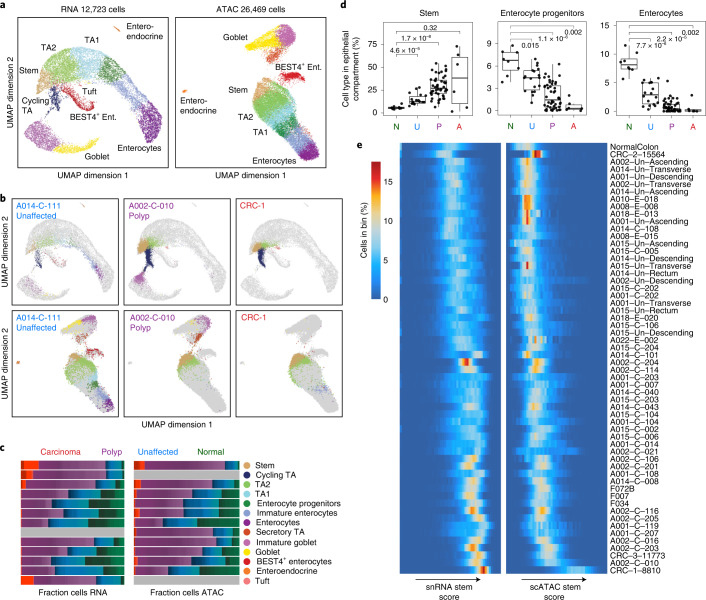
Stem-like features observed in epithelial cells. **a**, UMAP projection of snRNA-seq (left) and scATAC-seq (right) epithelial cells isolated from normal colon with cells colored by cell type. Colors for the cell types are defined in **c**. **b**, Projection of epithelial snRNA-seq (top) and scATAC-seq (bottom) cells from unaffected (left), polyp (center) and CRC (right) samples into the manifold of normal colon epithelial cells. Projected cells are colored by nearest normal cells in the projection and normal epithelial cells are colored gray. **c**, Fraction of each epithelial cell type isolated from normal (green), unaffected (blue), polyp (purple) and CRC (red) samples. Cell types are defined based on the identity of the nearest cell types when projecting epithelial cells into normal colon subspace. **d**, Boxplots depicting the fraction of cells within the epithelial compartment that are stem-like cells, enterocyte progenitors or enterocytes, divided by disease state. Abundances of each cell type in unaffected, polyp and CRC tissues are compared with their abundances in normal tissues with two-sided Wilcoxon testing and Bonferroni correction for multiple comparisons, and the resulting adjusted *P* values are listed in the plots. The boxplots are constructed with data from 8 normal samples, 18 unaffected samples, 48 polyp samples and 6 CRC samples. Boxplots represent the median, 25th percentile and 75th percentile of the data; whiskers represent the highest and lowest values within 1.5 times the interquartile range of the boxplot; and all points are plotted. **e**, Distribution of snRNA-seq and scATAC-seq stem scores in all epithelial cells in each sample. The rows represent individual samples and the columns represent 50 bins of stem scores from low to high for RNA (left) and ATAC (right). The heatmap is colored by the percentage of epithelial cells in each sample that are in a given bin of stem scores. A, adenocarcinoma; Ent., enterocyte; N, normal; P, polyp; TA, transit amplifying; U, unaffected FAP.

**Extended Data Fig. 6:**
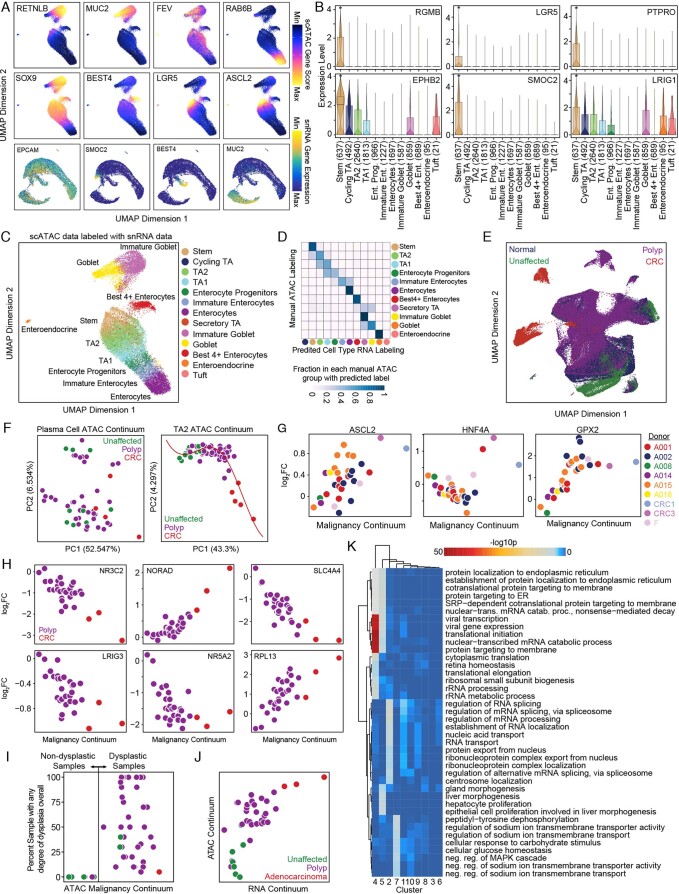
Characterization of normal colon epithelium and identification of changes along the malignancy continuum. (**a**) Upper 8 panels: UMAP projection of normal colon epithelial cells colored by scATAC gene activity scores of the epithelial marker genes *RETNLB* (immature goblet), *MUC2* (goblet), *FEV* (enteroendocrine), *RAB6B* (enterocyte), *SOX9* (stem), *BEST4* (Best4+ enterocyte), *LGR5* (stem), and *ASCL2* (stem). Lower 4 panels: UMAP projection of normal colon epithelial cells colored by expression of marker genes *EPCAM* (general epithelial), *SMOC2* (stem), *BEST4*, and *MUC2*. (**b**) Violin and boxplot representation of gene expression of stem marker genes by epithelial cell type. Asterisks indicate that gene expression is significantly upregulated when compared to all other cell types. Boxplots represent the median, 25th percentile, and 75th percentile of the data and whiskers represent the highest and lowest values within 1.5 times the interquartile range of the boxplots. (**c**) Labeling of scATAC-seq epithelial cells by nearest snRNA-seq cells following integration of the datasets with CCA. (**d**) Confusion matrix comparing annotation of scATAC cells using marker genes and labeling of scATAC cells with the nearest snRNA-seq cell following integration of scATAC and snRNA-seq datasets. (**e**) UMAP representation of snRNA-seq epithelial cells colored by disease state. (**f**) Results of computing the continuum on plasma cells and TA2 cells using the same method performed for stem cells. (**g**) Log_2_FC in expression of *ASCL2*, *HNF4A*, and *GPX2* in stem-like cells from each sample relative to stem-like cells in unaffected samples plotted against the malignancy continuum defined in 4D. Samples are colored based on the patient the sample was collected from. (H) Log_2_FC in expression of *NR3C2*, *NORAD*, *SLC4A4*, *LRIG3*, *NR5A2*, and *RPL13* as a function of malignancy continuum. Samples are colored based on if they are derived from polyps or CRCs. (**i)** Relationship between the malignancy continuum and percent of sample with any degree of dysplasia as determined by microscopic pathology. Samples are colored based on gross classification as a polyp (purple) or unaffected (green) tissue. Note that some samples classified as unaffected had dysplasia while some samples classified as polyps did not have dysplasia. **(i**) Relationship between the malignancy continuums defined from the scATAC and snRNA-seq datasets. Samples are colored based on gross classification as unaffected, polyp, or CRC. (**k**) Enrichment of gene ontology terms in clusters of differential RNA genes in Fig. 4.

We examined the epithelial cells that initially clustered by unaffected, polyp or CRC disease state (Fig. 1b,c and Extended Data Fig. 6e). To analyze these data, we first constructed RNA-seq and ATAC-seq references composed of normal epithelial colon cells collected from patients without FAP (Fig. 3a). We annotated cell types in this normal tissue using gene expression and gene activity scores of known marker genes (Extended Data Fig. 6a,b). A stem cell population with high expression and accessibility of *LGR5*, *SMOC2*, *RGMB*, *PTPRO*, *EPHB2* and *LRIG1* was evident (Extended Data Fig. 6b), as were goblet cells (*MUC2* high) and BEST4^+^ enterocytes (*BEST4* high). Following manual annotation, the snRNA-seq and scATAC-seq datasets were aligned with CCA^25,35^, and the scATAC cells were labeled based on the nearest snRNA-seq cells, which agreed with the manual annotations for 65% of cells, with mislabeled cells typically being labeled as the nearest cell type in the differentiation trajectory (Extended Data Fig. 6c,d).

**Extended Data Fig. 7:**
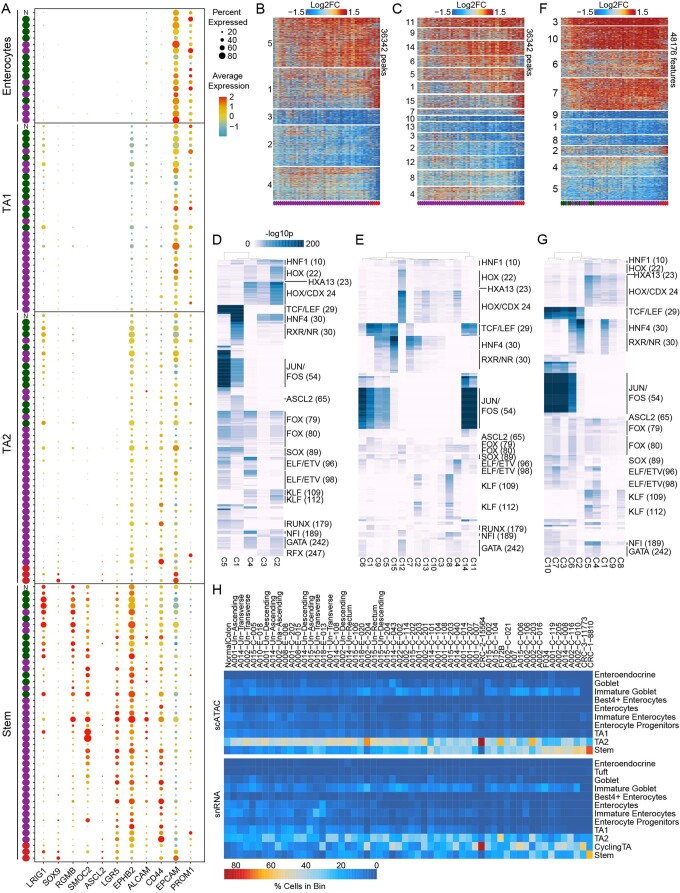
Motif enrichment in differential peaks with different numbers of k-means clusters. (**a**) Expression of intestinal stem cell and colon cancer stem cell marker genes in stem cells, TA2 cells, TA1 cells, and Enterocytes by sample. Samples are ordered by the malignancy continuum defined in Fig. 4. (B**, c**) Heatmaps of all peaks that were significantly differentially accessible (Wilcoxon test, padj≤0.05 & |log2FC | ≥1.5) in ≥2 samples. Samples are ordered along the x-axis by the malignancy continuum defined in Fig. 4. Peaks are k-means clustered into 5 (**b**) or 15 (**c**) clusters. (**d, e**) Hypergeometric enrichment of TF motifs in k-means clusters of peaks defined in B (**d**) and C (**e**). (**f**) Heatmap of all peaks that were significantly differentially accessible in ≥2 samples between stem cells from a given sample and normal colon stem cells. Samples are ordered along the x-axis by the malignancy continuum defined in Fig. 4. Peaks are k-means clustered into 10 groups. (**g**) Hypergeometric enrichment of TF motifs in k-means clusters of peaks defined in F. (**h**) Heatmap representation of cell types in each epithelial sample as determined by the nearest normal cell after projecting the cells into the normal LSI subspace.

We then projected the remaining cells into this normal subspace^25^, and found that epithelial cells from polyps and CRCs tend to project closer to stem cells and other immature cells along the normal differentiation trajectory, whereas cells from unaffected tissues projected relatively evenly throughout the epithelial compartment (Fig. 3b). We classified all epithelial cells based on the nearest normal cells in the projection and found that cells originating from polyps and CRC samples are enriched for stem-like epithelial cells and depleted for mature enterocytes, suggesting that epithelial cells increasingly demonstrate a stem-like phenotype during the transformation from normal to polyp (Fig. 3b–d and Extended Data Fig. 4a,b). We speculate that the populations of stem-like cells in the polyps and CRCs likely represent the ‘cancer’ stem cells in these tissues. Expression of previously described intestinal stem cell and colon cancer stem cell marker genes in these stem-like populations is discussed in detail in a Supplementary Note and Extended Data Fig. 7a.

To quantify the degree of stemness in individual cells within samples, we assigned scores quantifying stemness for each snRNA-seq and scATAC-seq cell and ordered samples by the distribution of stem scores within each sample (Methods and Fig. 3e). As expected, unaffected samples have generally lower stem scores. A number of polyps clustered near the unaffected tissues, suggesting that they are relatively benign. However, cells from most polyps and CRCs typically had higher stem scores, with some demonstrating a larger spread of stemness and others with much tighter distributions of stem scores, indicating that some polyps may be more heterogeneous. Similar results were observed when ordering samples based on the nearest normal cell type in the projection into the normal colon subspace (Methods and Extended Data Fig. 7h).

### Stem-like cells form a potential malignancy continuum

**Fig. 4:**
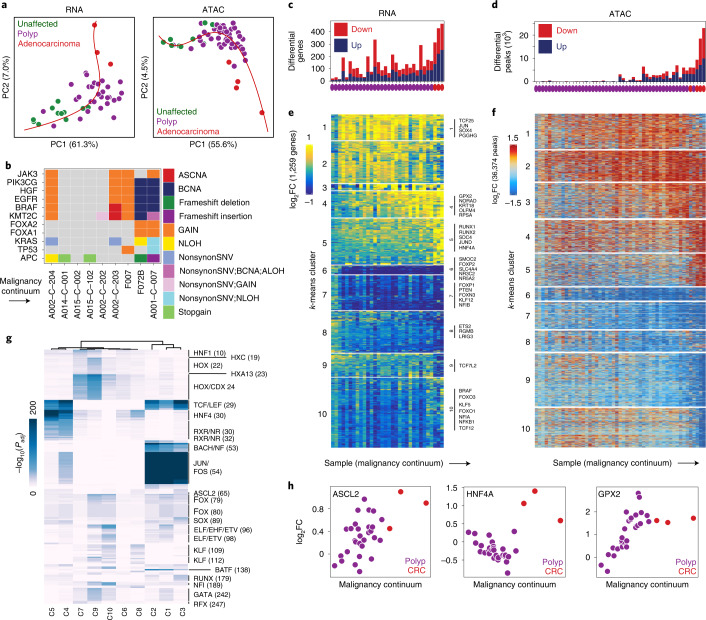
The regulatory trajectory of malignant transformation. **a**, Malignancy continuum for snRNA-seq (left) and scATAC-seq (right). Principal components were computed on the log_2_FC values between stem-like cells from each sample and normal colon stem cells for the set of peaks and genes that were significantly differential (Wilcoxon FDR ≤ 0.05 and |log_2_FC | ≥ 1.5 for peaks; MAST test for genes) in at least two samples. A spline was fit to the first two principal components (red) and samples were ordered based on their position along the spline. **b**, Genomic alterations in common driver genes ordered by the malignancy continuum. **c**,**d**, Number of significantly differential genes (MAST test) (**c**) and peaks (Wilcoxon test) (**d**) for each sample relative to all unaffected samples. **e**,**f**, Heatmap of all genes (**e**) and peaks (**f**) that were significantly differentially expressed (MAST test, *P*_adj_ ≤ 0.05 and |log_2_FC | ≥ 0.75) or accessible (Wilcoxon test, *P*_adj_ ≤ 0.05 and |log_2_FC | ≥ 1.5) in ≥2 samples. Samples are ordered along the *x* axis by the malignancy continuum defined in **d**. Genes and peaks are *k*-means clustered into ten groups. **g**, Hypergeometric enrichment of TF motifs in *k*-means clusters of peaks defined in **e**. **h**, log_2_FC in expression of *ASCL2*, *HNF4A* and *GPX2* in stem-like cells from each sample relative to stem-like cells in unaffected samples plotted against the malignancy continuum defined in **d**. Samples are colored based on if they are derived from polyps or CRCs.

We next compared the gene expression and chromatin accessibility of polyp and CRC stem-like cells with normal stem cells to identify the aberrant gene expression and regulatory programs in precancerous and cancerous lesions. After computing differential peaks between stem-like cells from each sample and cells from the nearest normal cell type, we computed the principal components of the log_2_FC for these peaks, then ordered samples by their position along a spline fit in this space (Fig. 4a), where position in ordering can be interpreted as position in a continuum from normal tissue to cancer. We generated a similar RNA trajectory using differential genes rather than differential peaks (Methods). The ordering of samples along the continua defined from the snRNA-seq and scATAC-seq datasets exhibited strong agreement (Extended Data Fig. 6j). This analysis suggests that differences in gene expression and chromatin accessibility between stem cells and these stem-like polyp cells follow a stereotyped progression from early to late polyp to invasive CRC.

To determine if this continuum is specific to the stem-like cells, which would be consistent with these cells being the only malignant cells in the samples, or if other epithelial cells also exhibit a continuum, which would be consistent with other cell types within the polyp being derived from cancer stem-like cells rather than normal cells, we performed the same analysis with TA2 cells (Extended Data Fig. 6f). We found that TA2 cells exhibit a similar continuum, suggesting that they continue to be derived from stem-like cells. When we perform a control analysis with plasma cells, which are not derived from cancer cells, we do not observe a similar continuum (Extended Data Fig. 6f). Comparison of the continuum with microscopic pathology and genomic alterations (Fig. 4b) is discussed in the Supplementary Information.

After computing the trajectory, we repeated the differential analysis using all unaffected samples rather than normal samples to increase the total number of patients and cells in the background group. We observe that the absolute number of significantly differential peaks and genes gradually increased along the malignancy continuum—with adenocarcinoma samples exhibiting the largest number of differential peaks and genes (Fig. 4c,d).

### Gene expression changes along the malignant continuum

**Extended Data Fig. 8:**
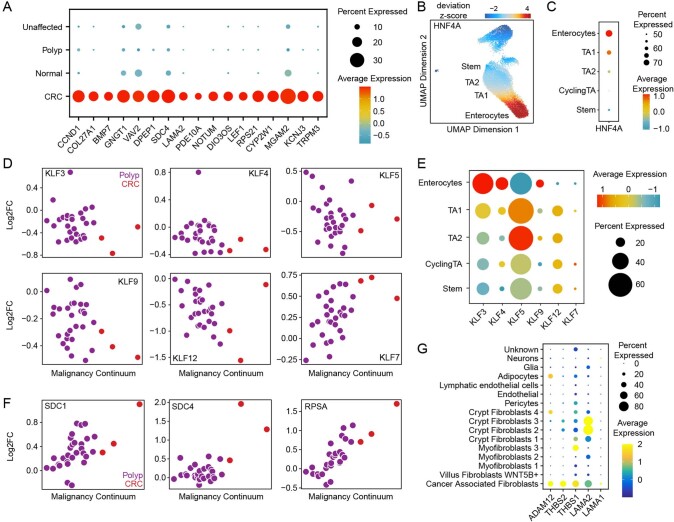
Epigenetic and transcriptomic changes along the malignancy continuum. (A) Dot plot representation of genes differentially expressed in CRC relative to polyps. (**b**) UMAP projection of normal colon epithelial cells colored by motif activity of HNF4A. **(c**) Dot plot representation of *HNF4A* expression in different normal colon epithelial subtypes. (**d**) Log_2_FC in expression of KLF TFs relative to unaffected colon as a function of position along the malignancy continuum. Samples are colored based on if they are from polyps (purple) or CRC (red). (**e**) Dotplot representation of the expression of KLF TFs in normal colon epithelial cells. (**f**) Log_2_FC in expression of *SDC1*, *SDC4*, and *RPSA* along the malignancy continuum. Samples are colored based on if they are from polyps (purple) or CRC (red). (**g**) Dotplot representation of the expression of selected ligands by different stromal cell types.

We examined gene expression changes along this malignancy continuum by selecting genes differentially expressed in at least two samples then clustering these genes into ten *k*-means clusters (Fig. 4e). These clusters correspond to groups of genes that become differentially expressed at distinct stages of malignant transformation. For example, clusters 1–4 comprise genes upregulated in stem-like cells in early-stage polyps when compared with unaffected stem cells. Members of cluster 4 include *OLFM4*, a marker of intestinal stem cells^36^, indicating that *OLMF4* expression increases in stem-like cells from polyps as they approach malignancy. Cluster 4 also includes GPX2, a glutathione peroxidase known to be upregulated in CRC that functions to relieve oxidative stress by reducing hydrogen peroxide, facilitating both tumorigenesis and metastasis^37^ (Fig. 4h). The upregulation is not donor dependent, and we observe the same trend across all donors in our study (Extended Data Fig. 6g). We observed translation Gene Ontology terms enriched in cluster 4 and splicing and RNA-processing Gene Ontology terms enriched in cluster 2 (Extended Data Fig. 6k). Clusters of genes that gradually reduce expression along the transition from normal colon to cancer (clusters 6–9) and genes specific to malignant transformation are discussed in a Supplementary Note and Extended Data Fig. 8a.

### Polyps demonstrate increased activity of TCF and LEF

To identify groups of polyps associated with invasive transformation, we clustered the 36,374 peaks significantly differential compared with the nearest unaffected cell type in at least two samples into ten *k*-means clusters (Fig. 4f), revealing five clusters that become more accessible and five clusters that become less accessible at different stages of the transition to cancer. To identify TFs driving chromatin accessibility changes in the transition from normal colon to CRC, we computed hypergeometric enrichment of motifs in each cluster of peaks from Fig. 4f (Fig. 4g) and ensured the stability of these results (Extended Data Fig. 7b–g).

TCF and LEF family motifs were enriched in all clusters that became more accessible across the malignancy continuum (clusters 1–5), consistent with the fact that loss of APC leads to β-catenin accumulation in the nucleus, which interacts with TCF and LEF TFs to drive WNT signaling^38–40^. This regulatory transformation is gradual across the malignant continuum—new peaks containing TCF and LEF motifs continue to open at all stages of colon cancer development, as does overall accessibility aggregated across TCF and LEF motifs, suggesting that WNT signaling gradually increases throughout this transformation, over and above what is observed in normal stem cell populations.

Cluster 3 peaks, which became more accessible in later-stage polyps and CRC, also exhibited enrichments of ASCL2 motifs (Fig. 4g). ASCL2 is a master regulator of intestinal stem cell fate, and induced deletion of ASCL2 leads to loss of LGR5^+^ intestinal stem cells in mice^41^. Consistent with a linkage between a more stem-like state in polyp epithelium and more advanced malignant continuum scores, *ASCL2* expression gradually increases as polyps approach malignant transformation (Fig. 4h), again indicative of a ‘super stem’-like phenotype, wherein master regulators of stem state are even more active than they are in normal stem cells.

Motifs lost along the malignancy continuum include HOX family motifs, KLF motifs and GATA motifs (Fig. 4g), and specific KLF TFs along the malignancy continuum are discussed in detail in a Supplementary Note and Extended Data Fig. 8d,e. Clusters 4 and 5 exhibit large accessibility increases only in CRC samples, and the greatest enrichment for HNF4A motifs (Fig. 4g). This observation suggests differential usage of HNF4A in polyps, where it decreases to drive WNT signaling, versus in CRC, where it is upregulated to drive cancer-specific accessibility differences (Supplementary Note and Extended Data Fig. 8b,c).

### Remodeling of cellular composition along malignant continuum

**Fig. 5:**
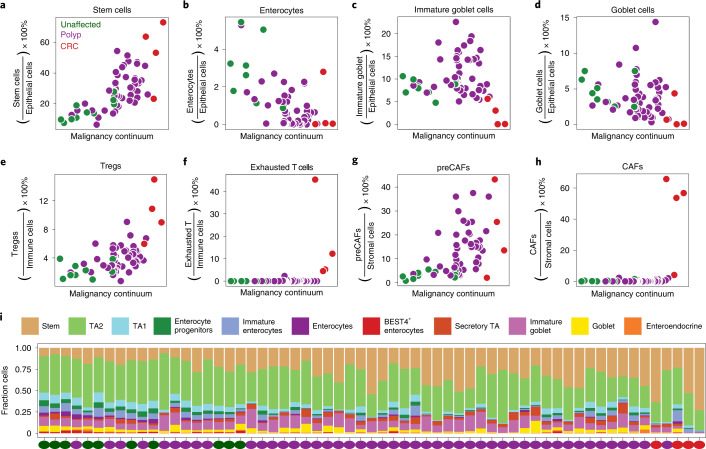
Dynamics of cell-type representation in malignant transformation. **a**–**h**, Fraction of cell type in each scATAC sample plotted against position of the sample in the malignancy continuum defined in Fig. 4d for stem-like cells (**a**), enterocytes (**b**), immature goblet cells (**c**), goblet cells (**d**), Tregs (**e**), exhausted T cells (**f**), preCAFs (**g**) and CAFs (**h**). Samples are colored based on if they are derived from unaffected tissues, polyps or CRCs. Fractions are computed by dividing the number of cells of a given cell type by the total number of cells in the compartment (epithelial versus immune versus stromal). **i**, Stacked boxplot representation of the fraction of epithelial cells of each cell type for each scATAC sample along the malignancy continuum.

We calculated the fractional contributions of each cell type to each sample as a function of position in the malignancy continuum, and found some cell types were highly correlated with progression along the malignancy continuum. For example, the fraction of stem cells within a sample gradually increases throughout malignant transformation (Fig. 5a,i). Similarly, the number of mature enterocytes decreases as polyps transform to carcinomas (Fig. 5b,i). Milo analysis revealed that neighborhoods of stem-like cells tend to be significantly more abundant at the end of the malignancy continuum (Extended Data Fig. 4b). In the secretory compartment, which primarily consists of immature and mature goblet cells, we observe a fractional increase in immature goblet cells in many polyps. In carcinomas we see a pervasive lack of differentiation into the secretory lineage, effectively eliminating immature and mature goblet cells (Fig. 5c,d,i). This observation is consistent with previous work reporting a depletion of goblet cells in nonmucinous colon adenocarcinomas^42^. Previous work has also found that knockout of MUC2 leads to the formation of more adenomas and carcinomas in mice^43^, suggesting that the loss of immature and mature goblet cells may even contribute to tumorigenesis.

Outside the epithelial compartment, we also observe changes in cellular composition across the transformation from unaffected to polyp to carcinoma. Within the stromal compartment, the fraction of preCAFs gradually increases, while CAFs only appear in CRCs (Fig. 5g,h). Within the immune compartment, Tregs are increased in the more malignant polyps and CRCs, while exhausted T cells only appear in CRCs (Fig. 5e,f and Extended Data Fig. 4b). Tregs are known to suppress the antitumor immune response and are typically present at high levels in the tumor microenvironment^44^. The gradual increase in Tregs may be a mechanism of immune evasion in precancerous polyps. We discuss possible cell–cell interactions between stromal and epithelial cells along the malignant continuum in a Supplementary Note and in Extended Data Fig. 8f,g.

### Comparing CRC DNA methylation changes with continuum accessibility

**Extended Data Fig. 9:**
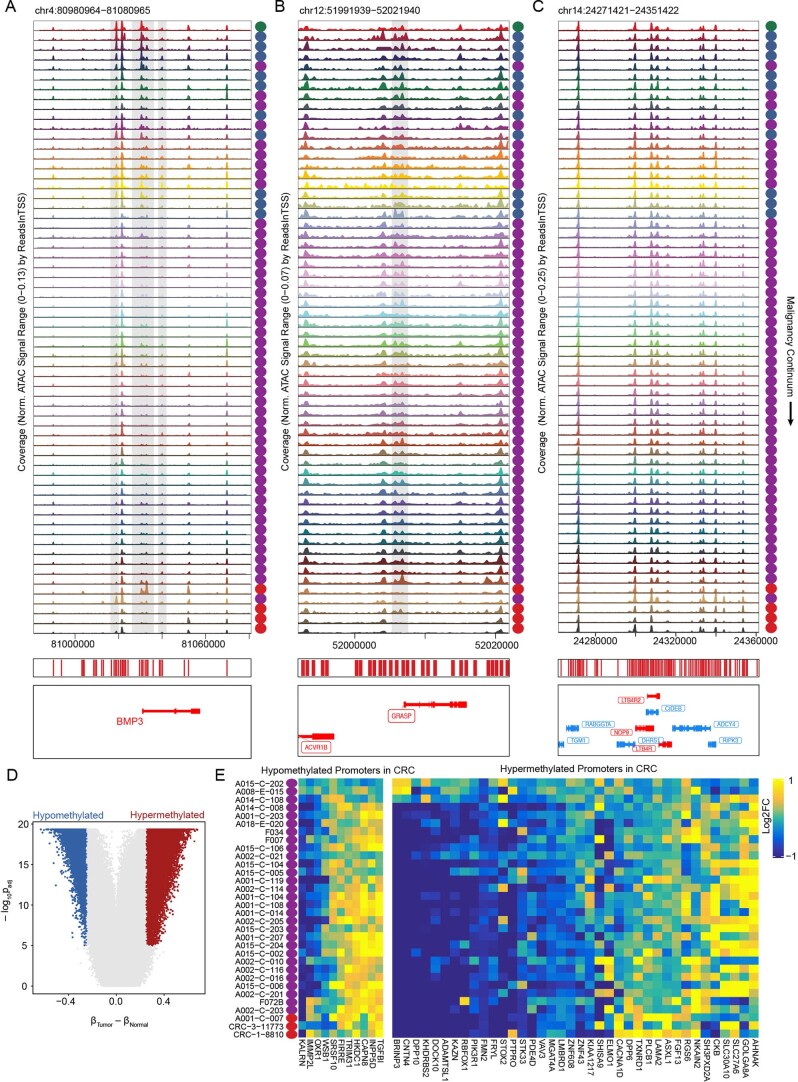
Accessibility changes in regions hypermethylated in CRC. (**a–c**) Accessibility tracks around *BMP3* (**a**), *GRASP* (**b**), and *CIDEB* (**c**), which are hypermethylated in CRC. (**d**) Adjusted p-value and mean difference in β-value cutoffs used to determine differentially methylated probes. P values were determined using the two-sided Wilcoxon test and were adjusted with the Benjamini-Hochberg method for multiple hypothesis testing. (**e**) Genes that were significantly differential along the malignancy continuum that also have differentially methylated probes within 500 bp of their TSS in TCGA 450 K methylation data. Genes are grouped into a heatmap of those with hypermethylated probes in their promoters and a heatmap of those with hypomethylated probes in their promoters.

Aberrant DNA methylation is a primary mechanism of tumorigenesis in CRC^45–47^, but the timing and extent to which methylation changes drive changes in chromatin accessibility before and during malignant transformation is not known. We identified differentially methylated probes between normal and CRC samples (Extended Data Fig. 9d) in The Cancer Genome Atlas (TCGA) DNA methylation data (Illumina 450K array)^48^. For the ~89,000 chromatin accessibility peaks from epithelial cells that overlap at least one 450K array probe, we determined how many overlapped at least one hypermethylated site, at least one hypomethylated site or no differentially methylated sites. We then divided the peaks into groups based on whether they were members of significantly upregulated or significantly downregulated clusters identified in Fig. 4h.

**Fig. 6:**
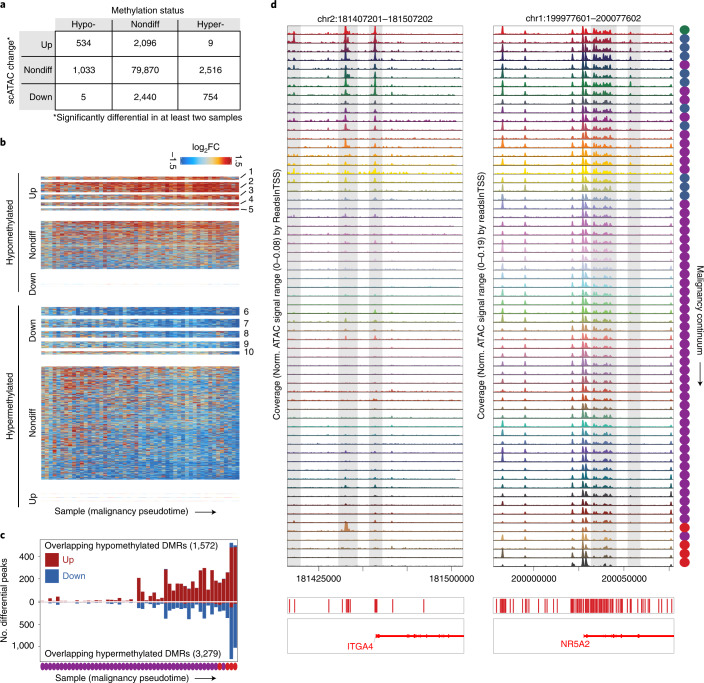
Integration of single-cell colon data with CRC methylation data reveals CRC DMRs with early changes in chromatin accessibility. **a**, Table relating the change in accessibility for peaks to the methylation status of Illumina 450K methylation probes they overlap. In total, ~89,000 peaks overlapped 180,000 450K probes. Peaks classified as up were members of clusters 1–5 in Fig. 4f and peaks classified as down were members of clusters 6–10 in Fig. 4f. **b**, Heatmaps of peaks overlapping hypomethylated (top) and hypermethylated (bottom) 450K probes in CRC. The heatmaps are split into peaks from more accessible and less accessible groups defined in Fig. 4h and peaks not included in Fig. 4h. For nondifferential (nondiff) peaks overlapping hypermethylated probes, ${{{P}}}\left( {\overline {{\mathrm{log}}_{2}{\rm{FC}}} < 0} \right) = 0.81$ and sign test *P* < 10^−50^. For nondifferential peaks overlapping hypomethylated peaks, ${{{P}}}\left( {\overline {{\mathrm{log}}_{2}{\rm{FC}}} > 0} \right) = 0.73$ and sign test *P* < 10^−50^. **c**, Number of significantly differential peaks overlapping hypomethylated or hypermethylated 450K probes for each sample. The total number of peaks overlapping hypermethylated and hypomethylated probes is listed in each plot. **d**, Accessibility tracks around *ITGA4* and *NR5A2*, which are hypermethylated in CRC. Tracks are ordered by position of the corresponding sample in the malignancy continuum defined in Fig. 4. DMR, differentially methylated region.

For peaks overlapping hypomethylated probes, approximately one-third (534) belonged to clusters that became significantly more accessible along the continuum, while <0.5% (5) became significantly less accessible (Fig. 6a). We saw similar correspondence for peaks overlapping hypermethylated probes, with approximately one-quarter (754) becoming less accessible, and <0.5% (9) becoming more accessible. Therefore, hypermethylation and hypomethylation in CRC nearly perfectly predict that accessibility at that site will either decrease or increase (respectively), or remain unchanged. In peaks not meeting the significance threshold, we still observe less aggregate accessibility within peaks overlapping hypermethylated probes and more accessibility when they overlap hypomethylated probes (Fig. 6b). However, we also observe that 79.4% (2,096) of significantly more accessible and 76.3% (2,440) of less accessible peaks overlap nondifferential probes, implying that a majority of chromatin accessibility changes are likely not driven by methylation.

We next plotted the number of differential peaks overlapping hypermethylated and hypomethylated probes across the malignancy continuum (Fig. 6c), and found that changes in chromatin accessibility that occur in regions that are ultimately differentially methylated in CRC accumulate along the transition from normal to cancer, with the greatest number observed in late-stage polyps and CRC.

Among regions that overlap hypermethylated probes in CRC that become less accessible in polyps are several previously reported cancer-specific hypermethylated loci^49^. For example, the promoter region and multiple distal regulatory elements near the *ITGA4* gene are accessible in normal colon, unaffected FAP colon and very early-stage polyps, but become closed early in the progression to CRC and remain closed even in low-grade polyps (Fig. 6d). The gene with the most nearby differential peaks overlapping hypermethylated probes in our dataset was *NR5A2*. Multiple peaks near this gene become less accessible along the malignancy continuum (Fig. 6d) and expression of *NR5A2* also gradually decreases along the malignancy continuum (Extended Data Fig. 6h). NR5A2 is a nuclear receptor that has been linked to a wide range of functions including inflammation and cell proliferation^50^. The hypermethylation, decrease in accessibility, and decrease in gene expression of *NR5A2* suggests that the pro-inflammatory state that may be triggered by the loss of NR5A2 might have a role in tumorigenesis.

Hypermethylated DNA regions in CRC have also been incorporated into CRC screening tests, including hypermethylation of the promoter regions of *BMP3* and *NDRG4* (ref. ^51^). We observe multiple distal elements around *BMP3* that become inaccessible in the middle of the malignancy continuum (Extended Data Fig. 9a). We observe many regions with a similar behavior: sharp increases or decreases in accessibility at a specific point along the malignancy continuum. We speculate that testing for accessibility, or methylation, at these loci may enable staging of polyps along the malignancy continuum. This approach also identifies methylation markers/loci (for example, *GRASP*, *CIDEB*) specific for malignant transformation in CRC (Extended Data Fig. 9b,c), and differential genes whose promoters overlap CRC methylation changes (Extended Data Fig. 9e).

## Discussion

Strategies to identify individuals in a premalignant stage, where interventions might be highly efficacious, promise tractable means to prevent cancer deaths. However, most previous work profiling genetic, epigenetic and transcriptomic changes that occur in malignancy has focused on advanced tumors rather than premalignant lesions. Our single-cell atlas of colon cancer tumorigenesis fills this gap by identifying key changes in chromatin accessibility, gene expression and tissue composition that occur along this transformation, and provides a wealth of potential targets for prevention, diagnosis and treatment of malignancy.

**Extended Data Fig. 10:**
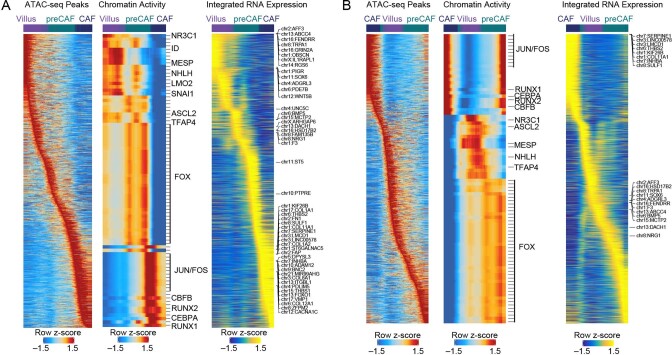
Trajectory analysis of preCAFs and CAFs. (**a**) Changes in most variable peaks, TF motif activity scores, and gene expression along the trajectory from villus fibroblasts to preCAFs to CAFs. (**b**) Changes in most variable peaks, TF motif activity scores, and gene expression along a control trajectory from CAFs to villus fibroblasts to preCAFs.

Analysis of both the composition and cell state of precancerous epithelial cells revealed that an increasing fraction of epithelial cells occupy a stem-like state as polyps approach malignancy, but these cells also exhibit underlying dysfunctional epigenetic and gene expression programs distinct from normal stem cells. We also identify compositional and cell state changes of noncancerous cells, including fibroblasts and immune cells, which may influence the tumor microenvironment to drive cancer progression. Within the fibroblast compartment, we identified a population of fibroblasts enriched in polyps and adenocarcinoma samples that shared many features with CAFs. We discuss the possibility that preCAFs may be on the path of becoming CAFs in a Supplementary Note and in Extended Data Fig. 10.

Gene expression changes along the malignancy continuum implicate mechanisms of cancer initiation and nominate diagnostic and therapeutic targets. We find that expression of *GPX2*, which encodes a glutathione peroxidase, is gradually upregulated across the transformation from normal tissue to malignancy. Even in premalignant tissues, *GPX2* is upregulated, suggesting that its role in reducing the oxidative environment may be needed for progression along this continuum. As a result, we speculate that expression of *GPX2* could serve as a marker for the degree of polyp malignancy, and that inhibitors of GPX enzymes—such as tiopronin^52^—may be relevant treatment strategies for CRC or premalignant lesions.

We identified several TFs associated with chromatin accessibility changes as polyps transition to malignancy. In stem-like cells obtained from polyps, we observe that many TCF and LEF motifs inaccessible in normal intestinal stem cells become accessible, suggesting that WNT signaling increases along the malignant continuum over and above that of normal stem cells. The final step in cancer formation is malignant transformation, and we identify diverse changes in chromatin accessibility, gene expression and tissue composition associated with this transformation (Figs. 4 and 5 and Extended Data Figs. 4, 6 and 8). Our data indicate that HNF4A may be a key regulator of malignant transformation—as both the expression of this gene becomes upregulated and chromatin regions containing HNF4A motifs become accessible only after the transformation to CRC. In normal colon, HNF4A motifs are more accessible in mature enterocytes than stem cells, but HNF4A is upregulated in CRC stem cells and drives chromatin accessibility changes not observed in normal stem cells.

Previous work identified epigenetic factors associated with CRCs, and DNA methylation state markers are at the core of widely adopted screening tests for CRC^51^. While it is unknown when in tumorigenesis these methylation state changes occur, our dataset revealed that these regions become inaccessible in the middle of the continuum. Because we observe that hypermethylated regions are strongly decreased in their accessibility and hypomethylated regions are strongly enriched for increased accessibility, our dataset can be used to stratify differentially methylated regions expected to be present early in the malignant continuum, those expected to be present later in the continuum and those expected to be present only in CRC, opening the possibility for a stage-specific molecular screening.

Currently, clinical guidelines for the timing and frequency of follow-up endoscopic screening after polypectomy depend on the size and degree of dysplasia of the polyps removed^53^. We found variability in the degree of transformation in polyps with the same dysplasia classification. This work presents a strategy to order premalignant polyps by their degree of malignancy, which we speculate may be useful for staging polyps and assessing clinical risk. Perhaps the most straightforward approach to this type of staging is to use the fraction of Tregs or stem-like cells (through immunohistochemical labeling of cell-type-specific markers such as LGR5) as a proxy for position along the malignancy continuum (Fig. 4 and Extended Data Figs. 6 and 8). However, determining if these molecular features correlate with patient outcomes will require substantial future clinical investigation.

This work demonstrates that adenomatous polyps traverse a strikingly consistent epigenetic and transcriptional trajectory as they progress to CRC. These results lead us to question if a similar relatively uniform molecular phenotypic trajectory is common to other premalignant lesions that precede other cancers, or if other pre-cancers might traverse multiple diverse paths on the way to malignant transformation. Further, we might also ask if CRCs continue to follow a consistent pathway to metastasis or if multiple distinct pathways are taken once malignant transformation occurs. We anticipate that similar single-cell integrative methods can be deployed to answer these questions.

## Methods

### Experimental methods

#### Description of FAP donors

We collected samples from the following groups of patients: FAP (eight patients), routine colonoscopy screening (one patient), sporadic CRC (four patients) and healthy controls (two patients). This study was approved by the Stanford Institutional Review Board and informed consent was obtained from all patients. All patients with FAP had clinical FAP. FAP tissue was collected at the time of partial or full colectomies for four patients and during screening colonoscopies for four patients. From non-FAP patients, one sporadic polyp was obtained during a standard colonoscopy as part of routine screening, four sporadic CRCs were obtained from the Stanford Tissue Bank and nine normal tissue samples were collected from brain-dead organ donors under consent.

Patient-matched normal colon mucosa, polyps and adenocarcinomas were flash frozen in liquid nitrogen at time of collection and stored at −80 °C. One polyp (A002-C-202) and one adenocarcinoma (A001-C-007) were embedded in optimal cutting temperature compound before storage at −80 °C. Polyps were scored by a board-certified pathologist for presence of dysplasia, including low or high grade with corresponding percentages. A small number of polyps (seven polyps) were exhausted for molecular assays so pathology reads were not obtained. The isolation of nuclei was accomplished using the OmniATAC protocol^54^ or the S2 Singulator (four samples) and is described further in the Supplementary methods.

Following nuclei dissociation, scATAC-seq targeting 9,000 cells per sample was performed using Chromium Next GEM Single Cell ATAC Library & Gel Bead Kit v.1.1 (10x Genomics, 1000175) and snRNA-seq targeting 9,000 cells per sample was performed using Chromium Next GEM Single Cell 3′ Reagent Kits v.3.1 (10x Genomics, 1000121). Samples were sequenced on a NovaSeq 6000 Illumina sequencer (Supplementary methods).

### Analytical methods

#### scATAC-seq: running Cell Ranger

Initial processing of scATAC-seq data was performed using the Cell Ranger ATAC Pipeline (https://support.10xgenomics.com/single-cell-atac/software/pipelines/latest/what-is-cell-ranger-atac, v.1.2.0) by first running cellranger-atac mkfastq to demultiplex the bcl files and then running cellranger-atac count to generate scATAC fragments files. These fragments files were loaded into R (v.3.6.1) using the createArrowFiles function in ArchR (v.0.9.5)^55^. Quality control metrics were computed for each cell, and cells with TSS enrichments less than 4 were filtered out for all samples. Cells were also filtered based on the number of unique fragments sequenced using a cutoff defined for each sample. As the sequencing depth sometimes differed between samples, it was necessary to assign this fragment cutoff as a sample-specific parameter. The sample-specific cutoffs ranged from 1,500 to 10,000, with the most common cutoff being 3,000 fragments per cell.

#### scATAC-seq: doublet removal and initial clustering

After generating arrow files for each sample, an ArchR project containing all samples was created. Doublets were simulated using the ArchR function addDoubletScores with *k* = 10. Cells with the highest probability of being doublets were removed using the ArchR function filterDoublets with a filterRatio of 1.2. Following initial doublet removal, sample-wise quality control statistics including TSS enrichment and fragments per cell were recomputed (Extended Data Fig. 1a). As part of construction of the arrow files, a tile matrix consisting of reads in 500-bp tiles was constructed. This tile matrix was used as input to compute an iterative latent semantic indexing (LSI) dimensionality reduction using the addIterativeLSI function in ArchR with a total of 2 iterations, a clustering resolution of 0.2 following the first iteration, 25,000 variable features, 30 dimensions and sampling 50,000 cells. Following dimensionality reduction, initial clustering was performed using ArchR’s addClusters, which is a wrapper for Seurat’s FindClusters function^35^, using a resolution of 1.7 and sampling 50,000 cells for clustering (remaining cells were then grouped into clusters based on the nearest cells included in the clustering). We next ran addUMAP on the IterativeLSI dimensionality reduction with 30 nearest neighbors and a minimum distance of 50.

ArchR computes gene activity scores for each gene, which are a function of the accessibility within and around a given gene body and can be used as a proxy for gene expression. After initial dimensionality reduction and clustering of all cells in our dataset, we examined gene activity scores of known marker genes for cell types expected to be present in epithelial, stromal or immune cells and divided cells from our dataset into three groups (immune, stromal or epithelial) for downstream analysis.

#### scATAC-seq: analysis of immune compartment

After subsetting the dataset to include only immune cells, we repeated dimensionality reduction and clustering. The iterative LSI dimensionality reduction was computed using addIterativeLSI with 2 iterations, 25,000 variable features, sampleCellsPre set to NULL, dimensions 1–30 and a clustering resolution of 0.2 for the initial iteration. Clusters were then determined with addClusters in ArchR using the Seurat method, a resolution of 1.7 and nOutlier set to 50. The UMAP dimensionality reduction was then computed using addUMAP with 30 nearest neighbors, a minimum distance of 0.5 and the cosine metric. We next examined gene activity scores of known marker genes and identified three small clusters with gene activity scores for marker genes that were not consistent with these clusters consisting of a single high-quality immune cell subtype. As a result, these clusters were thought to be likely doublets, and were removed before additional analysis. We note this approach to removing likely doublet clusters is commonly employed for single-cell datasets and a similar approach has recently been applied to remove doublets in a large snRNA-seq dataset on colon cells^22^. This is further discussed in the Supplementary methods section ‘Removal of possible doublet clusters’. Following this additional doublet removal step, the dimensionality reduction and clustering steps were repeated using identical parameters.

Multiple approaches were taken to annotate the scATAC data. First, gene activity scores of known marker genes for different immune populations expected to be present in the scATAC data were examined for the different scATAC clusters. Marker genes included *PAX5*, *MS4A1*, *CD19*, *IGLL5* and *VPREB3* for B cells; *TPSAB1*, *HDC*, *CTSG*, *CMA1*, *KRT1*, *IL1RAPL1* and *GATA2* for mast cells; *KLRF1*, *SH2D1B* and *SH2D1B* for natural killer cells; *SSR4*, *IGLL5*, *IGLL1* and *AMPD1* for plasma cells; *CD14* for monocytes; *CD3D*, *CD3E*, *CD3G*, *CD8A*, *CD8B*, *TBX21*, *IL7R*, *CD4*, *CD2*, *BATF*, *TNFRSF4*, *FOXP3*, *CTLA4* and *LAIR2* for T cells and T cell subtypes; and *FOLR2*, *FABP3* and *PLA2G2D* for macrophages. This approach led to unambiguous identification of most clusters in our dataset. While annotating the clusters, some clusters were labeled with the same annotation if they consisted of cells of the same subtype. We next integrated our scATAC data with multiple snRNA-seq datasets using ArchR’s addGeneIntegrationMatrix function, and then labeled scATAC cells based on the nearest snRNA-seq cells. This included large, high-quality single-cell RNA-seq (scRNA-seq) data from cells isolated from normal colon and patients with ulcerative colitis^22^. This dataset contains slightly different populations of cells (for example, no exhausted T cells) so was insufficient to be used for annotation of our data in isolation. However, we observed good overall agreement between the marker gene-based annotations of our scATAC data and the annotations obtained when labeling our scATAC cells with the nearest scRNA-seq cell in the Smillie et al. dataset (Extended Data Fig. 1h)^22^. We also integrated our data with the labeled snRNA-seq data produced in this study, which also produced good agreement with our initial manual labeling (Extended Data Fig. 1i). Ultimately, our final annotations are the result of both initial annotation with known marker genes and refinement and validation of our clusters by integrating our scATAC data with multiple scRNA-seq datasets and labeling our scATAC cells with the nearest scRNA cells from these datasets.

#### scATAC-seq: evidence of T cell exhaustion

We aimed to determine if T cell exhaustion could be detected in early stages of the transition to carcinoma or only later after malignant transformation. We subclustered the T cells in our dataset, projected these cells into a UMAP and colored the cells by disease state of the tissue of origin or their cell-type annotations (Supplementary methods and Extended Data Fig. 3a,b). We supported the presence of exhausted T cells in four different ways. First, we examined gene activity scores for exhausted T cell markers including *BATF*, *CTLA4*, *PDCD1* and *TOX*, and found that they were high in one cluster (Extended Data Fig. 3c). Second, we used a previously published snRNA-seq dataset that contained exhausted T cells from basal cell carcinoma^15^ to identify exhausted T cells within the subclustering of T cells by aligning the datasets with CCA and labeling our cells by the closest RNA-seq profiles as described above^15^ (Extended Data Fig. 3e). Third, we identified exhausted T cell-specific regulation by identifying differentially accessible peaks (Wilcoxon test) relative to CD8^+^ T cells (Extended Data Fig. 3f). Similar to CD8^+^ T cells, exhausted T cells exhibit a high level of accessibility around the CD8 locus, suggesting that they are likely CD8^+^ (Extended Data Fig. 3g). Peaks more accessible in exhausted T cells were enriched for NR4A, RUNX, CEFB, JUN, FOS and BATF family motifs, many of which are known drivers of T cell exhaustion^56^. Fourth, we computed chromVAR deviations, which also show that BATF and NR4A2 motifs tend to be more accessible in exhausted T cells (Extended Data Fig. 3d). Together, these four lines of evidence identified the same population of exhausted T cells, which are observed in CRC samples only, demonstrating that this specific immunological dysfunction seems to be unique to invasive cancer samples.

#### scATAC-seq: construction of normal epithelial reference and projection into normal reference

To generate a normal epithelial reference, epithelial cells (as defined by gene activity scores of known epithelial marker genes) from nine samples taken from two genetically normal donors were selected. Next, we followed a dimensionality reduction and clustering protocol similar to what is described above for the immune and stromal cell types. An iterative LSI dimensionality reduction was performed with addIterativeLSI with 4 iterations; 15,000 variable features; and clustering resolutions of 0.1, 0.1 and 0.2 following the first three iterations. Clusters were then defined with a resolution of 2 and a likely doublet cluster was removed, defined based on no clear accessibility around marker genes and the cluster consisting of cells from only a subset of the samples. The dimensionality reduction was then repeated with the above parameters. To simplify the clustering, we ran harmony batch correction (v.1.0)^57^ on the IterativeLSI dimensionality reduction and clustered the cells with a resolution of 2.5. This did not substantially change the structure of the data, but facilitated cell-type annotation. We note that cluster annotations would be similar without this step and we show all annotations on the UMAP generated from the IterativeLSI dimensionality reduction. Clusters were annotated based on gene activity scores at known marker genes. Marker genes include *DCLK1*, *HTR3C*, *HTR3E* and *B4GALNT4* for tuft cells; *KLK1*, *ITLN1*, *WFDC2* and *CLCA1* for immature goblet cells; *MUC2*, *TFF1*, *FCGBP* and *TBX10* for goblet cells; *CA1* for immature enterocytes, *RAB6B* for enterocytes; *CRYBA2* and *SCGN* for enteroendocrine cells; *BEST4*, *CA7*, *OTOP2* and *OTOP3* for BEST4^+^ enterocytes; and *SMOC2*, *RGMB*, *LGR5* and *ASCL2* for stem cells. These marker genes are supported by the literature and many have been previously shown to be specific markers for these cell types in scRNA-seq data^22^. ChromVar deviations for the normal epithelial reference were computed in ArchR as described above.

After generation of a normal epithelial reference, we next aimed to project diseased cells into this subspace, as has previously been done for placing diseased cells isolated from mixed-phenotype acute leukemia samples into the hematopoietic hierarchy^25^. To accomplish this, we started with the tile matrix for epithelial cells from a given sample. We then selected the features from this tile matrix that were used in the final LSI iteration for the normal epithelial cells. We computed the inverse document frequency using the number of rows and columns from the initial LSI computation and performed the same singular value decomposition (SVD) transformation as was done in the final LSI iteration. After projecting each cell into this IterativeLSI subspace, we identified the 25 nearest neighbor cells with get.knnx in R. Cell-type annotations were then assigned for each cell based on the most common cell-type annotation of the 25 nearest neighbors. Please see the Supplementary methods for details on definition of the peak set and determination of differential peaks.

#### scATAC-seq: definition of malignancy continuum

To compute the malignancy continuum, we computed differential peaks between stem cells from all polyp, unaffected and CRC samples (71 samples) in our dataset and normal stem cells (nine samples). Unaffected samples from the same region of the colon in the same patient were merged to ensure that there was a sufficient number of stem cells to compute differentials, which left 68 total unaffected, polyp and CRC samples. After computing differentials individually for each sample, we selected the set of peaks that was significantly differential in at least two samples (Wilcoxon FDR ≤ 0.05 and |log_2_FC | ≥ 1.5 in ≥2 samples). We then constructed the matrix of log_2_FC values for this set of significant peaks in all samples. The principal components of these differentials were computed with prcomp in R and a spline was fit to the first two principal components. For each sample, we then identified the nearest point on the spine (minimum Euclidean distance), and the samples were ordered based on the position of the nearest point on the spline fit. We only included scATAC samples with at least 250 stem cells in the malignancy continuum, as we have higher confidence in the differentials computed with a larger number of cells. The threshold of 250 stem cells excluded seven samples, leaving a total of 61 samples in the continuum.

#### scATAC-seq: identification of differential peaks and enriched motifs along the malignancy continuum

To identify differential peaks along the malignancy continuum, unaffected samples were used as a background because we observed relatively small differences between unaffected and normal tissues, and because including more samples allowed us to better match potentially biasing features such as read depth and TSS enrichment when computing differentials. A few unaffected samples were found to have dysplasia on microscopic pathology and as a result were not included in the background when computing these differentials. We first identified differential peaks between stem-like cells in each polyp and CRC sample and stem cells from all unaffected tissues. Peaks that were significantly differential in at least two samples (Wilcoxon FDR ≤ 0.05 and |log_2_FC | ≥ 1.5 in ≥2 samples) were clustered into ten groups with *k*-means clustering using the kmeans function in R with iter.max set to 500. Following clustering of the differential peaks into ten groups, hypergeometric enrichment of clustered TF motifs^58^ (which were downloaded from https://jeffgranja.s3.amazonaws.com/ArchR/Annotations/Vierstra-Human-Motifs.rds) within those ten groups was calculated using the ArchR function peakAnnoEnrichment. We note that this same analysis was done using normal stem cells as the background for differential testing and produced very similar results (Extended Data Fig. 4b,c).

#### snRNA-seq: initial processing

Initial processing of snRNA-seq data was done with the Cell Ranger Pipeline (https://support.10xgenomics.com/single-cell-gene-expression/software/pipelines/latest/what-is-cell-ranger, v.3.1.0) by first running cellranger mkfastq to demultiplex the bcl files and then running cellranger count. Since nuclear RNA was sequenced, data were aligned to a pre-messenger-RNA reference.

#### snRNA-seq: doublet removal and initial clustering

After running Cell Ranger, the filtered_feature_bc_matrix produced by Cell Ranger was read into R with the Seurat (v.3.1.1)^35^ function Read10X. The data were filtered to remove cells with fewer than 400 unique genes per cell or greater than 4,000 genes per cell. DoubletFinder (v.2.0.3)^59^ was run for each sample using principal components 1–20. nExp was set to 0.08 × nCells^2^/10,000, pN to 0.25 and pK to 0.09. Cells classified as doublets were then removed before additional analysis.

After running DoubletFinder, the remaining cells from all samples were merged into a single Seurat object and cells with greater than 10,000 counts per cell or greater than 5% mitochondrial RNA were removed. The data were then processed with Seurat’s standard pipeline. First, NormalizeData was run using the method LogNormalize and scale.factor of 10,000. Variable features were identified with Seurat’s findVariableFeatures using the vst method and 20,000 features. ScaleData was then run on all genes and principal components were computed with RunPCA. The cells were then clustered using Seurat’s FindNeighbors with dimensions 1–20 and FindClusters with a resolution of 1.0. Expression of marker genes in the resulting clusters was then used to label clusters as epithelial, stromal or immune for downstream analysis. Dimensionality reduction, clustering and annotation of immune and stromal compartments is discussed in the Supplementary methods.

#### snRNA-seq: construction of normal epithelial reference and projection into normal reference

To analyze the epithelial compartment, we first constructed a normal epithelial reference using epithelial cells from normal colon taken from genetically normal donors. One normal sample was excluded from the construction of the reference to serve as a test set to project into the normal subspace. We started with data normalized with Seurat’s normalizeData function. Next, the iterative LSI dimensionality reduction was computed using four total iterations, following a procedure outlined previously^25,60^. For each iteration, the mitochondrial, ribosomal and HLA genes were filtered out and, from the remaining genes, the top 1,600 most variable genes were identified. We then computed the term frequency–inverse document frequency (TF-IDF) transformation on these genes and performed SVD on the transformed matrix, and provided dimensions 1–8 of this reduction as input to Seurat’s shared nearest neighbor clustering with resolution of 0.1. We summed the individual clusters single cells, computed the log(counts per million) transformation with ‘edgeR::cpm(mat,log = TRUE,prior.count = 3)’ and then found the top 1,600 variable genes across the clusters. A TF-IDF transformation was then computed on these variable genes and an SVD was then performed on the transformed matrix. Dimensions 1–8 were retained and clusters were identified using the Seurat functions findNeighbors and findClusters, but with an increased resolution of 0.2. This process was repeated a total of four times with a resolution of 0.4 after the third iteration. After the final dimensionality reduction, we found that the fifth LSI component was highly correlated with the sample of origin (by biserial correlation), so removed that dimension and used dimensions 1–4 and 6–8 for additional downstream analysis. Using the iterative LSI approach with only eight dimensions allowed us to denoise the data and limit batch effect, which was useful for the projections.

This final LSI dimensionality reduction was provided as input to compute a UMAP representation of the data and the cells were clustered using a resolution of 1.0. The resulting clusters were then annotated based on expression of known marker genes. The projection of cells into the LSI subspace defined for normal colon epithelial cells was done following the procedure described previously^25^. Briefly, when computing the TF-IDF transformation on normal colon epithelial cells, we stored the colSums, rowSums and SVD. To project cells from additional samples into this subspace, we first zero out rows based on the initial TF-IDF rowSums. We next calculated the term frequency by dividing by the column sums and computed the inverse document frequency from the previous TF-IDF transformation. These were then used to compute the new TF-IDF. The resulting TF-IDF matrix was projected into the previously defined SVD. Cells were classified by identifying their 25 nearest neighbors in the LSI subspace using get.knnx in R and then classifying the cell as the most common annotation for those 25 nearest neighbors.

#### snRNA-seq: definition of malignancy continuum and determination of differential genes along continuum

Similar to scATAC, differential genes were computed between stem cells from each sample and stem cells from normal colon. To compute differential genes for the snRNA-seq dataset, the Seurat function FindMarkers was used with ident.1 set as the sample of interest, ident.2 set as the background_sample, min.pct = 0, logfc.threshold = 0, min.cells.feature = 0, max.cells.per.ident = 300 and Model-based Analysis of Single-cell Transcriptomics (MAST) used as the differential test^61^. We merged unaffected samples from the same region to provide more cells for computing differentially expressed genes.

For computing the snRNA-seq malignancy continuum, an analogous process to the one used for ATAC was carried out with the following minor differences: (1) differential genes rather than differential peaks were used, (2) significance cutoffs for including a gene were MAST *P*_adj_ ≤ 0.05 and |log_2_FC | ≥ 0.5 in ≥2 samples and (3) we required there to be at least 100 snRNA-seq cells in a group to compute differentials. Following determination of the RNA malignancy continuum, we computed differential genes between each polyp and CRC sample (32 of 49 samples with at least 100 cells) against all unaffected samples as was done for scATAC and plotted the set of differential genes with MAST *P*_adj_ ≤ 0.05 and |log_2_FC | ≥ 0.75 in ≥2 samples in the heatmap in Fig. 4.

#### Analysis of DNA methylation data

Analysis of TCGA 450K methylation data was done with TCGABioloinks (v.2.12.6)^62^. Data for normal colon and colorectal adenocarcinoma were downloaded using the function GDCquery with project = c(‘TCGA-COAD’), data.category = ‘DNA Methylation’, legacy = FALSE, platform = c(‘Illumina Human Methylation 450’) and sample.type = c(‘Primary solid Tumor’,’Solid Tissue Normal’). Differentially methylated probes between normal and colorectal adenocarcinoma samples were computed using TCGAanalyze_DMR with a *P* value cutoff of 10^−5^ and a mean difference in *β* value cutoff of 0.25 to determine significance. Overlaps between DNA methylation probes and our peak set were identified with the GenomicRanges function FindOverlaps in R.

#### Supplementary methods

Please see the Supplementary methods for additional methodologic details, including details on CODEX multiplex imaging.

### Reporting summary

Further information on research design is available in the Nature Research Reporting Summary linked to this article.

## Online content

Any methods, additional references, Nature Research reporting summaries, source data, extended data, supplementary information, acknowledgements, peer review information; details of author contributions and competing interests; and statements of data and code availability are available at 10.1038/s41588-022-01088-x.

## Supplementary information

Supplementary Information Supplementary text and methods.

Reporting Summary

Supplementary Tables Supplementary Table 1. Donor metadata including age, sex and race/ethnicity. Supplementary Table 2. Sample-specific metadata including donor, location, storage method and pathology. Supplementary Table 3. HTAN Biospecimen IDs for HTAN data used in this study. Supplementary Table 4. HuBMAP Sample IDs for HuBMAP scATAC data used in this study. Supplementary Table 5. HuBMAP Sample IDs for HuBMAP snRNA-seq data used in this study.

## Data Availability

Sequencing data have been deposited in the Gene Expression Omnibus (GEO) with the accession code GSE201349. Original data generated in this study are also available on the Human Tumor Atlas Network (HTAN) Data Portal (unaffected FAP tissues, polyps and CRCs; https://data.humantumoratlas.org/ under the HTAN Stanford Atlas) and the HuBMAP data portal (normal colon tissues; https://portal.hubmapconsortium.org/ under the Stanford TMC). Unique IDs for accessing the HTAN datasets are listed in Supplementary Table 3 and unique IDs for accessing the HuBMAP datasets are listed in Supplementary Tables 4 and 5. Receptor ligand pairs from the Fantom5 database were downloaded from https://fantom.gsc.riken.jp/5/suppl/Ramilowski_et_al_2015/ (ref. ^63^). Clustered TF motifs can be downloaded from https://www.vierstra.org/resources/motif_clustering#downloads (ref. ^58^). Seurat objects for previously published single-cell colon data were downloaded from https://github.com/cssmillie/ulcerative_colitis (ref. ^22^). Counts matrices and T cell annotations for cells from BCC are available on GEO with accession number GSE123813 (ref. ^15^). TCGA DNA methylation data can be downloaded from the GDC data portal (https://portal.gdc.cancer.gov/)^48^.

## Code availability

Scripts for processing the single-cell data, generating the malignancy continuum and analyzing the WGS data are available on GitHub (https://github.com/winstonbecker/scCRC_continuum).

## References

[1] Weinstein JN (2013). The Cancer Genome Atlas Pan-Cancer analysis project. Nat. Genet..

[2] International Cancer Genome Consortium et al. (2010). International network of cancer genome projects. Nature.

[3] The ICGC/TCGA Pan-Cancer Analysis of Whole Genomes Consortium. Pan-cancer analysis of whole genomes. *Nature* **578**, 82–93 (2020).

[4] Fodde R, Smits R, Clevers H (2001). APC, signal transduction and genetic instability in colorectal cancer. Nat. Rev. Cancer.

[5] Aoki K, Taketo MM (2007). Adenomatous polyposis coli (APC): a multi-functional tumor suppressor gene. J. Cell Sci..

[6] Leslie A, Carey FA, Pratt NR, Steele RJC (2002). The colorectal adenoma-carcinoma sequence. Br. J. Surg..

[7] Vogelstein B (1988). Genetic alterations during colorectal-tumor development. N. Engl. J. Med..

[8] Fearon ER, Vogelstein B (1990). A genetic model for colorectal tumorigenesis. Cell.

[9] Mori Y (1992). Somatic mutations of the APC gene in colorectal tumors: mutation cluster region in the APC gene. Hum. Mol. Genet..

[10] Logan CY, Nusse R (2004). The Wnt signaling pathway in development and disease. Annu. Rev. Cell Dev. Biol..

[11] Schatoff EM, Leach BI, Dow LE (2017). Wnt signaling and colorectal cancer. Curr. Colorectal Cancer Rep..

[12] Groden J (1991). Identification and characterization of the familial adenomatous polyposis coli gene. Cell.

[13] Galiatsatos P, Foulkes WD (2006). Familial adenomatous polyposis. Am. J. Gastroenterol..

[14] Rozenblatt-Rosen O (2020). The Human Tumor Atlas Network: charting tumor transitions across space and time at single-cell resolution. Cell.

[15] Yost KE (2019). Clonal replacement of tumor-specific T cells following PD-1 blockade. Nat. Med..

[16] Dann E, Henderson NC, Teichmann SA, Morgan MD, Marioni JC (2022). Differential abundance testing on single-cell data using *k*-nearest neighbor graphs. Nat. Biotechnol..

[17] de Vries NL (2020). High-dimensional cytometric analysis of colorectal cancer reveals novel mediators of antitumour immunity. Gut.

[18] Vinay DS (2015). Immune evasion in cancer: mechanistic basis and therapeutic strategies. Semin. Cancer Biol..

[19] Blank CU (2019). Defining ‘T cell exhaustion’. Nat. Rev. Immunol..

[20] Jiang Y, Li Y, Zhu B (2015). T-cell exhaustion in the tumor microenvironment. Cell Death Dis..

[21] Powell DW, Pinchuk IV, Saada JI, Chen X, Mifflin RC (2011). Mesenchymal cells of the intestinal lamina propria. Annu. Rev. Physiol..

[22] Smillie CS (2019). Intra- and inter-cellular rewiring of the human colon during ulcerative colitis. Cell.

[23] Karpus ON (2019). Colonic CD90^+^ crypt fibroblasts secrete semaphorins to support epithelial growth. Cell Rep..

[24] Kabiri Z (2014). Stroma provides an intestinal stem cell niche in the absence of epithelial Wnts. Development.

[25] Granja JM (2019). Single-cell multiomic analysis identifies regulatory programs in mixed-phenotype acute leukemia. Nat. Biotechnol..

[26] Koliaraki V, Pallangyo CK, Greten FR, Kollias G (2017). Mesenchymal cells in colon cancer. Gastroenterology.

[27] Tommelein J (2015). Cancer-associated fibroblasts connect metastasis-promoting communication in colorectal cancer. Front. Oncol..

[28] Karagiannis GS (2012). Cancer-associated fibroblasts drive the progression of metastasis through both paracrine and mechanical pressure on cancer tissue. Mol. Cancer Res..

[29] Lee K-W, Yeo S-Y, Sung CO, Kim S-H (2015). Twist1 is a key regulator of cancer-associated fibroblasts. Cancer Res..

[30] Puré E, Blomberg R (2018). Pro-tumorigenic roles of fibroblast activation protein in cancer: back to the basics. Oncogene.

[31] Fang S (2019). Clinical significance and biological role of cancer-derived Type I collagen in lung and esophageal cancers. Thorac. Cancer.

[32] Asano K (2017). Stromal versican regulates tumor growth by promoting angiogenesis. Sci. Rep..

[33] Unterleuthner D (2020). Cancer-associated fibroblast-derived WNT2 increases tumor angiogenesis in colon cancer. Angiogenesis.

[34] Aizawa T (2019). Cancer‐associated fibroblasts secrete Wnt2 to promote cancer progression in colorectal cancer. Cancer Med..

[35] Stuart T (2019). Comprehensive integration of single-cell data. Cell.

[36] Flier LGvander (2009). OLFM4 is a robust marker for stem cells in human intestine and marks a subset of colorectal cancer cells. Gastroenterology.

[37] Emmink BL (2014). GPx2 suppression of H_2_O_2_ stress links the formation of differentiated tumor mass to metastatic capacity in colorectal cancer. Cancer Res..

[38] Cadigan KM, Waterman ML (2012). TCF/LEFs and Wnt signaling in the nucleus. Cold Spring Harb. Perspect. Biol..

[39] Morin PJ (1997). Activation of β-catenin-Tcf signaling in colon cancer by mutations in β-catenin or APC. Science.

[40] Korinek V (1997). Constitutive transcriptional activation by a β-catenin-Tcf complex in APC^−/−^ colon carcinoma. Science.

[41] van der Flier LG (2009). Transcription factor achaete scute-like 2 controls intestinal stem cell fate. Cell.

[42] Leow CC, Romero MS, Ross S, Polakis P, Gao W-Q (2004). Hath1, down-regulated in colon adenocarcinomas, inhibits proliferation and tumorigenesis of colon cancer cells. Cancer Res..

[43] Velcich A (2002). Colorectal cancer in mice genetically deficient in the mucin Muc2. Science.

[44] Plitas G, Rudensky AY (2020). Regulatory T cells in cancer. Annu. Rev. Cancer Biol..

[45] Ashktorab H, Brim H (2014). DNA methylation and colorectal cancer. Curr. Colorectal Cancer Rep..

[46] Hinoue T (2012). Genome-scale analysis of aberrant DNA methylation in colorectal cancer. Genome Res..

[47] Lengauer C, Kinzler KW, Vogelstein B (1997). DNA methylation and genetic instability in colorectal cancer cells. Proc. Natl Acad. Sci. USA.

[48] Cancer Genome Atlas Network. (2012). Comprehensive molecular characterization of human colon and rectal cancer. Nature.

[49] Barault L (2018). Discovery of methylated circulating DNA biomarkers for comprehensive non-invasive monitoring of treatment response in metastatic colorectal cancer. Gut.

[50] Fernandez-Marcos PJ, Auwerx J, Schoonjans K (2011). Emerging actions of the nuclear receptor LRH-1 in the gut. Biochim. Biophys. Acta.

[51] Imperiale TF, Ransohoff DF, Itzkowitz SH (2014). Multitarget stool DNA testing for colorectal-cancer screening. N. Engl. J. Med..

[52] Hall MD (2014). Inhibition of glutathione peroxidase mediates the collateral sensitivity of multidrug-resistant cells to tiopronin. J. Biol. Chem..

[53] Gupta S (2020). Recommendations for follow-up after colonoscopy and polypectomy: a consensus update by the US Multi-Society Task Force on Colorectal Cancer. Gastrointest. Endosc..

[54] Corces MR (2018). The chromatin accessibility landscape of primary human cancers. Science.

[55] Granja JM (2021). ArchR is a scalable software package for integrative single-cell chromatin accessibility analysis. Nat. Genet..

[56] Satpathy AT (2019). Massively parallel single-cell chromatin landscapes of human immune cell development and intratumoral T cell exhaustion. Nat. Biotechnol..

[57] Korsunsky I (2019). Fast, sensitive and accurate integration of single-cell data with Harmony. Nat. Methods.

[58] Vierstra J (2020). Global reference mapping of human transcription factor footprints. Nature.

[59] McGinnis CS, Murrow LM, Gartner ZJ (2019). DoubletFinder: doublet detection in single-cell RNA sequencing data using artificial nearest neighbors. Cell Syst..

[60] Aran D (2019). Reference-based analysis of lung single-cell sequencing reveals a transitional profibrotic macrophag. Nat. Immunol..

[61] Finak G (2015). MAST: a flexible statistical framework for assessing transcriptional changes and characterizing heterogeneity in single-cell RNA sequencing data. Genome Biology.

[62] Colaprico A (2016). TCGAbiolinks: an R/Bioconductor package for integrative analysis of TCGA data. Nucleic Acids Res..

[63] Ramilowski JA (2015). A draft network of ligand-receptor-mediated multicellular signalling in human. Nat. Commun..

