# Supplementary material for: Single-cell analyses define a continuum of cell state and composition changes in the malignant transformation of polyps to colorectal cancer
Source: Nat Genet. 2022 Jun 20;54(7):985–95. doi: 10.1038/s41588-022-01088-x (PMC9279149; doi:10.1038/s41588-022-01088-x)
Supplement: Supplementary file 1 — Supplementary text and methods. [file 41588_2022_1088_MOESM1_ESM.pdf]

nature portfolio

https://doi.org/10.1038/s41588-022-01088-x

Supplementary information

Single-cell analyses define a continuum of cell state and composition changes in the malignant transformation of polyps to colorectal cancer

In the format provided by the authors and unedited

SUPPLEMENTARY INFORMATION FOR *SINGLE-CELL ANALYSES DEFINE A CONTINUUM OF CELL STATE AND COMPOSITION CHANGES IN THE MALIGNANT TRANSFORMATION OF POLYPS TO COLORECTAL CANCER*

I. SUPPLEMENTARY NOTES

NOMINATION OF REGULATORY ELEMENTS DRIVING WNT2 EXPRESSION

We identified a regulatory element ~50 kb away from the *WNT2* TSS that is most accessible in CAFs whose accessibility is highly correlated to gene expression of WNT2, leading us to speculate that this regulatory element may drive higher *WNT2* expression in these cells (Fig. 2B). Another peak, ~25KB from the *WNT2* TSS, is also more accessible in CAFs, but accessibility at this peak was less correlated with gene expression in our linked datasets.

EXPRESSION OF STEM CELL MARKER GENES BY STEM-LIKE POPULATIONS

To further characterize the polyp and CRC stem-like cells, we examined expression of intestinal stem cell and colon cancer stem cell marker genes in these stem-like populations. A number of markers for intestinal stem cells have been identified, including LGR5, SMOC2, RGMB, SOX9, and LRIG1 [1]. Colon cancer stem cells have also been reported to express LGR5, and have been proposed to be CD133+, EPCAM^{high}, CD44+, CD166+, ALDH+, and EphB2^{high} [1]. Additionally, ASCL2 has been shown to be a master regulator for intestinal stem cells [2]. Among these markers, we find that *LRIG1* and *RGMB* are expressed primarily in stem cells from normal and unaffected tissues, *LGR5*, *EPHB2*, and *ALCAM/CD166* are highly expressed in stem-like cells from normal, unaffected, polyp, and CRC samples, and *CD44*, *EPCAM*, *ASCL2*, and *SOX9* are highest expressed in stem-like cells from polyps and CRCs (Extended Data Fig. 7A). *LGR5* expression is specific to stem-like cells in all samples, and has low or no expression in other cell types, further supporting that projecting diseased cells into the normal manifold effectively identifies stem cell populations.

COMPARISON OF MALIGNANCY CONTINUUM TO MICROSCOPIC PATHOLOGY AND GENOMIC ALTERATIONS

The order of the stem cells may serve as a proxy for the progression of a polyp toward malignant transformation. We obtained microscopic pathology on the majority of our polyps and they were graded as containing no dysplasia, low-grade dysplasia, or high-grade dysplasia. Nearly all of the polyps in our study were graded as containing low-grade dysplasia; however, our molecular findings suggest these polyps represent a large diversity of phenotypes on a relatively continuous spectrum from normal epithelia to invasive cancer. We found that samples with no dysplasia on microscopic pathology are the earliest samples on the malignancy continuum. Notably, two samples that we classified as polyps during sample collection were found to have no dysplasia on pathology review, and these samples were the earliest polyps in the malignancy continuum. Further, two unaffected samples were found to have low grade dysplasia, and these unaffected samples were the unaffected samples the furthest along the malignancy continuum. Beyond this binary separation of samples with dysplasia or no dysplasia, the degree of transformation in each polyp was not highly correlated with the fraction of cells classified as dysplastic as determined

from microscopic pathology (Figure S6I), although this dysplastic fraction measure is not used as a clinically relevant metric for assessing polyp severity.

To probe the genetic features that may drive progression along this continuum, we performed whole genome sequencing on 8 polyp samples and 1 CRC and examined somatic mutations in colon cancer driver genes, including *APC*, *KRAS*, *TP53*, and *BRAF* (Fig. 4B). Although these samples followed a stereotyped phenotypic continuum, they exhibited diverse genetic changes and the appearance of different driver mutations did not occur at specific positions along the continuum, suggesting that many of the same phenotypic changes may occur from a range of genetic alterations.

DECREASES IN GENE EXPRESSION ALONG THE MALIGNANT CONTINUUM

Multiple gene clusters gradually reduce expression in the transition from normal colon to cancer (clusters 6–9). Members of these clusters include *NR3C2*, which codes for the mineralocorticoid receptor (Extended Data Fig. 6H). Low expression of *NR3C2* has been observed in multiple cancers and is associated with poor prognosis. Knockdown of *NR3C2* in hepatocellular carcinoma cells leads to an increase in β-catenin expression [3]. Therefore, decreased expression of *NR3C2* in CRC may be another means to increase expression of WNT target genes and drive proliferation in polyps and CRC. LRIG3, a transmembrane protein recently reported to repress cell motility and metastasis in CRC [4] also gradually decreases in expression across the malignancy continuum (Extended Data Fig. 6H). Genes specific to malignant transformation are discussed in a Supplementary Note.

GENE EXPRESSION CHANGES SPECIFIC TO MALIGNANT TRANSFORMATION

While we observe many genes that are gradually upregulated or downregulated along the malignancy continuum, we also wanted to highlight genes specific to malignant transformation as they may represent the transcriptomic alteration necessary for invasion (Figure S8A). Many of the genes specific to malignant transformation are previously described markers for CRC that we find to be specific to CRC and not highly expressed in adenomas. For example, BMP7, a secreted signaling factor, is specifically upregulated in CRC and has been shown to correlate with metastasis and poor prognosis in CRC [5]. Another gene found to be specifically expressed in CRC was DPEP1, a zinc-dependent metalloprotease involved in glutathione and leukotriene metabolism, that has previously been hypothesized as a marker of high grade intraepithelial neoplasia [6].

PEAKS WITH KLF MOTIFS ARE LOST IN MALIGNANT TRANSFORMATION

We next examined the clusters of peaks that became less accessible along the malignancy continuum. Among these clusters were groups of peaks that are less accessible in most polyps (cluster 6) and groups of peaks that only become less accessible in CRC (cluster 9). Motifs enriched in these clusters included HOX family motifs, KLF motifs, and GATA motifs, amongst others. Enrichment of KLF factors is notable as some KLF factors, such as KLF4, have been shown to be tumor suppressors in CRC [7]. Indeed, loss of accessibility at peaks containing KLF motifs occurs across the malignancy continuum (cluster 8–10; Figure 4G).

We observe that many KLF factors, such as *KLF3*, *KLF4*, *KLF5*, *KLF9*, and *KLF12* decrease in expression along the malignancy continuum (Figure S8D). In normal colon differentiation, some KLF factors contribute to maintaining a proliferative state, while others are associated with epithelial differentiation [8]. We find that these same KLF factors that decrease in expression in stem cells along the malignancy continuum are less expressed in normal stem cells relative to more mature epithelial cells, suggesting that these factors may drive differentiation in normal colon (Figure S8E). As a result, loss of these factors in stem-like cells along the malignancy continuum may prevent differentiation of stem-like cells in polyps and CRC. *KLF7* was an exception to this trend, as expression of *KLF7* increased in stem-like cells along the malignancy continuum (Figure S8D). However, unlike the other factors, *KLF7* was more highly expressed in normal colon stem and cycling cells (Figure S8E) compared with normal enterocytes. This indicates that, unlike other KLF factors, overexpression of *KLF7* in polyps and CRC may also prevent differentiation of stem-like cells.

HNF4A DRIVES CANCER SPECIFIC CHROMATIN ACCESSIBILITY

Clusters 4 and 5 exhibit large accessibility increases only in CRC samples, and the greatest enrichment for HNF4A motifs (Fig. 4G). Interestingly, HNF4A expression decreases across polyps along the malignancy continuum, but is then greatly over expressed following transformation to CRC. These observations may reconcile previous seemingly contradictory observations regarding the role of the TF HNF4A in the development of CRC. On one hand, conditional knockout of HNF4A in adult mice leads to increased proliferation in the crypts and increased WNT signaling, suggesting that HNF4A may be a tumor suppressor gene [9]. Similar to this finding, we observe that motifs of HNF4A are most accessible and that HNF4A is highest expressed in mature epithelial cells in normal colon, suggesting that HNF4A is at least associated with less cell proliferation (Extended Data Figs. 8B,C). However, other studies have shown that knockdown of HNF4A in CRC cell lines inhibits growth [10] and that HNF4A is overexpressed in CRC [11]. In our data, HNF4A is gradually lost in polyps, likely leading to an increase in WNT signaling and proliferation, which is consistent with the finding of increased proliferation when HNF4A is overexpressed in normal colon. However, when the transformation to carcinoma occurs, HNF4A becomes overexpressed and begins to drive accessibility of cancer-specific peaks. When we examine GO enrichment in RNA cluster 5, which consists of genes most upregulated in CRC samples, we found enrichment of GO terms for hepatocyte proliferation and epithelial cell proliferation involved in liver morphogenesis (Extended Data Fig. 6K).

NOMINATION OF STROMAL–EPITHELIAL LINKS IN CANCER DEVELOPMENT

To further investigate possible interactions between stromal and epithelial cells, we examined if stem-like epithelial cells in polyps and CRCs expressed receptors that are targets of ligands expressed by CAFs. To do this we first computed potential ligands that were differentially upregulated in CAFs relative to other stromal cell types. We then used the Fantom5 database[12] to generate a list of possible receptors for the upregulated ligands. We tested if the differential expression of these receptors in stem-like cells was highly correlated with position along the malignancy continuum. Among the receptors that were most correlated with position along the malignancy continuum were the syndecan family proteins, SDC1 and SDC4 (Figure S8F). Syndecan proteins function as coreceptors for growth factors, matrix proteins, cytokines, and

chemokines and have been suggested to both promote or suppress cancer [13]. One possible mechanism by which these proteins could contribute to cancer formation is through signals from the developing tumor microenvironment. We found that a number of genes that are upregulated in CAFs are known to interact with syndicans. For example, SDC4 interacts with ADAM12 [14], which was previously identified in tumor-associated stroma [15], and is highly expressed in CAFs in our dataset (Figure S8G). Thrombospondin proteins also bind syndicans [16], and are highly expressed in CAFs.

Another transmembrane receptor that gradually increased in expression along the malignancy continuum was RPSA (Figure S8F), which acts as a cell surface receptor for lamins and is thought to facilitate invasion and metastasis by altering cancer cell interactions with the extracellular matrix [17]. We also observed high expression of lamins in CAFs (Figure S8G), which may provide increased lamins to bind RPSA receptors on cancer stem cells. Together, these analyses nominate pairs of ligands and receptors that become increasingly expressed along the malignancy continuum by CAFs and stem-like cells respectively, suggesting mechanisms by which CAFs may work with precancerous cells to contribute to tumorigenesis.

SPECULATION ON A TRAJECTORY FROM preCAFs to CAFs

The observation that, when compared to other populations of fibroblasts, preCAFs had relatively higher accessibility at many known CAF marker genes led us to hypothesize that these preCAFs may be on the path towards becoming CAFs. To further map out the changes in these populations of fibroblasts, we constructed a trajectory between villus fibroblasts, preCAFs, and CAFs and examined changes in peaks and chromVAR activity scores along this putative trajectory. When we plot the most variable peaks, we observe a relatively monotonic opening and closing of peaks along this trajectory (Figure S10A), which is not observed in a control analysis of a trajectory from CAFs to villus fibroblasts to preCAFs (Figure S10B). Chromatin accessibility activity levels of DNA motifs (quantified using chromVAR deviation z-scores) along this trajectory show increased activity of FOX family transcription factors in the intermediate states, that is followed by increased activity of JUN, FOS, CEBP, and RUNX1 motifs in CAFs (Figure S10). The smooth accessibility and expression changes along this putative trajectory are consistent with the hypothesis that normal colon fibroblasts, such as villus fibroblasts, develop into preCAFs and eventually CAFs; however, proving this would likely require lineage tracing experiments, and it is possible that CAFs develop through a different pathway. Alternatively, preCAFs may just represent dysregulation of a normal fibroblast subtype that supports the increasingly stem-like cells in the polyp.

REFERENCES

1. Heijden, M. van der, van der Heijden, M. & Vermeulen, L. Stem cells in homeostasis and cancer of the gut. *Molecular Cancer* vol. 18 (2019).
2. van der Flier, L. G. *et al.* Transcription factor achaete scute-like 2 controls intestinal stem cell fate. *Cell* **136**, 903–912 (2009).

3. Yang, C. *et al.* MicroRNA-766 promotes cancer progression by targeting NR3C2 in hepatocellular carcinoma. *FASEB J.* **33**, 1456–1467 (2019).
4. Zeng, K. *et al.* LRIG3 represses cell motility by inhibiting slug via inactivating ERK signaling in human colorectal cancer. *IUBMB Life* **72**, 1393–1403 (2020).
5. Motoyama, K. *et al.* Clinical significance of BMP7 in human colorectal cancer. *Ann. Surg. Oncol.* **15**, 1530–1537 (2008).
6. Eisenach, P. A. *et al.* Dipeptidase 1 (DPEP1) is a marker for the transition from low-grade to high-grade intraepithelial neoplasia and an adverse prognostic factor in colorectal cancer. *Br. J. Cancer* **109**, 694–703 (2013).
7. Wei, D., Kanai, M., Huang, S. & Xie, K. Emerging role of KLF4 in human gastrointestinal cancer. *Carcinogenesis* **27**, 23–31 (2006).
8. Kim, C.-K., He, P., Bialkowska, A. B. & Yang, V. W. SP and KLF Transcription Factors in Digestive Physiology and Diseases. *Gastroenterology* **152**, 1845–1875 (2017).
9. Cattin, A.-L. *et al.* Hepatocyte nuclear factor 4alpha, a key factor for homeostasis, cell architecture, and barrier function of the adult intestinal epithelium. *Mol. Cell. Biol.* **29**, 6294–6308 (2009).
10. Schwartz, B. *et al.* Inhibition of colorectal cancer by targeting hepatocyte nuclear factor-4α. *International Journal of Cancer* vol. 124 1081–1089 (2009).
11. Cancer Genome Atlas Network. Comprehensive molecular characterization of human colon and rectal cancer. *Nature* **487**, 330–337 (2012).
12. Ramilowski, J. A. *et al.* A draft network of ligand-receptor-mediated multicellular signalling in human. *Nat. Commun.* **6**, 7866 (2015).
13. Sayyad, M. R. *et al.* Syndecan-1 facilitates breast cancer metastasis to the brain. *Breast Cancer Res. Treat.* **178**, 35–49 (2019).
14. Thodeti, C. K. *et al.* ADAM12/syndecan-4 signaling promotes beta 1 integrin-dependent cell spreading through protein kinase Calpha and RhoA. *J. Biol. Chem.* **278**, 9576–9584 (2003).

15. Peduto, L. *et al.* ADAM12 is highly expressed in carcinoma-associated stroma and is required for mouse prostate tumor progression. *Oncogene* **25**, 5462–5466 (2006).

16. Barbouri, D. *et al.* Syndecans as modulators and potential pharmacological targets in cancer progression. *Front. Oncol.* **4**, 4 (2014).

17. Ménard, S., Tagliabue, E. & Colnaghi, M. I. The 67 kDa laminin receptor as a prognostic factor in human cancer. *Breast Cancer Res. Treat.* **52**, 137–145 (1998).

II. SUPPLEMENTARY METHODS

Tissue Dissociation and Nuclei Isolation:

All protocols used to generate snRNA-seq data on the 10x Chromium platform, including sample prep, library prep, instrument, and sequencing settings, can be found on the 10x Genomics website at: https://support.10xgenomics.com/single-cell-gene-expression. The isolation of nuclei was accomplished using the OmniATAC protocol [18]. Dissociation of nuclei was carried out entirely on wet ice. 40-60mg of flash-frozen tissue was placed into 2ml dounce tissue grinders containing 1ml HB (Lysis) Buffer (1.0341x HB Stable solution, 1M DTT, 500 mM Spermidine, 150mM Spermine, 10% NP40, cOmplete Protease Inhibitor, Ribolock), gently triturated, then allowed to thaw for 5 minutes in the solution. After 5 minutes, tissue was dounced 10 times with pestle A and 20 times with pestle B, or until there was no resistance from either pestle. Sample was filtered through a 40um cell strainer (Falcon; 352340) and resulting homogenate transferred to a pre-chilled 2ml LoBind tube. Samples were spun in a 4°C fixed angle centrifuge for 5 minutes at 350 RCF to pellet nuclei. After spinning, all but 50ul of supernatant was removed. 350ul HB was added to the nuclei pellet for a total volume of 400ul. Nuclei were gently resuspended with a wide bore pipet. One volume of 50% Iodixanol (60% OptiPrep [Sigma Aldrich; D1556], Diluent Buffer [2M KCl, 1M MgCl2, 0.75M Tricine-KOH pH 7.8], Water) was added and gently triturated. Next, 600ul of 30% Iodixanol was carefully layered under the 25% mixture. Finally, 600ul of 40% Iodixanol was layered under the 30% mixture. Sample was then spun in a 4°C swinging bucket centrifuge for 20 min at 3,000 RCF. Upon completion of the spin, a band of nuclei was visible. Supernatant was aspirated down to within 200-300ul of the nuclei band. The nuclei band was then collected at 200ul and transferred to a fresh 1.5ml tube. Sample was diluted with one volume (200ul) Resuspension Buffer (1x PBS, 1% BSA, 0.2u/uL Ribolock). Nuclei concentration was determined using the Countess II FL Automated Cell Counter (ThermoFisher; AMQAF1000).

In addition to isolating nuclei with the omni-ATAC dounce method, we used the Singulator from S2 Genomics to isolate nuclei for 4 samples (2 normal and 2 polyp) from patient A002. The same buffers (lysis, wash, resuspension) were used for both the manual omni-ATAC dounce and S2 Singulator methods. HB buffer was prepared following the OmniATAC protocol, with the addition of 15ul Ribolock per 1mL HB buffer to help maintain RNA integrity. Approximately 50-70mg tissue in small chunks were placed into the Nuclei Isolation Cartridge. We ran the Extended Nuclei Isolation Singulator program which includes disruption, 5 minute incubation, disruption, filtration (150um and 40um filters) and buffer rinse of the cartridge. Following dissociation, nuclei were spun at 300g for 5 minutes. Supernatant was removed and nuclei were resuspended in Resuspension Buffer (noted above). Nuclei were counted using the Countess Automated Cell Counter (ThermoFisher). The total dissociation time for 4 samples was approximately 1 hour, as one sample can be run at a time on the S2 Singulator. Data generated from nuclei dissociated with the Omni-ATAC protocol and S2 genomics exhibited excellent agreement (Figures S1J–S1L).

Nuclei isolated with both methods were immediately used for downstream processing. Subsequent downstream GEM barcoding, cDNA construction, and gene expression library construction were performed according to the Chromium Next GEM Single Cell 3' Reagent Kits

v3.1 (10x Genomics, 1000121) and Chromium Next GEM Single Cell ATAC Library & Gel Bead Kit v1.1 (10x Genomics, 1000175). Average time from dissociation to generating barcoded GEMs for snRNA-seq was 30-45 minutes.

Single-cell assay for transposase-accessible chromatin using sequencing (scATAC-seq)
scATAC-seq targeting 9,000 cells per sample was performed using Chromium Next GEM Single Cell ATAC Library & Gel Bead Kit v1.1 (10x Genomics, 1000175) and Chromium Next GEM Chip H (10x Genomics, 1000161). Each sample library was uniquely barcoded and quantified by qPCR using a PhiX Control v3 (Illumina, FC-110-3001) standard curve. Libraries were then pooled and loaded on a NovaSeq 6000 Illumina sequencer (1.4 pM loading concentration, 50 × 8 × 16 × 49 bp read configuration) and sequenced targeting an average of 25,000 reads per cell.

Single-nuclear whole transcriptome sequencing (snRNA-seq):
snRNA-seq targeting 9,000 cells per sample was performed using Chromium Next GEM Single Cell 3' Reagent Kits v3.1 (10x Genomics, 1000121) and Chromium Next GEM Chip G Single Cell Kit (10x Genomics, 1000120). Each sample library was uniquely barcoded and quantified by qPCR using a PhiX Control (Illumina, FC-110-3001) standard curve. Libraries were pooled and loaded onto NovaSeq 6000 Illumina sequencer (Read 1 = 28bp, i7 index=8bp, i5 index=0bp, Read 2=91bp read configuration) and sequenced targeting an average of 20,000 reads per cell.

Single-cell ATAC-seq: Sub Clustering of T-cells
We next selected all T-cells present in our scATAC dataset to further explore differences in the T-cell subtypes. Using only the T-cells in our dataset, we ran addIterativeLSI with the same parameters as the full immune data. The cells were clustered using addClusters with a resolution of 2.5 and nOutlier of 50. Impute weights were computed for the T-cell subset and imputed gene activity scores of T-cell marker genes were plotted on the T-cell UMAP with the ArchR function plotEmbedding. The scATAC T-cell dataset was integrated with a previously published dataset consisting of T-cells from BCC [19] using addGeneIntegrationMatrix as described above.

T-cell specific peaks were called using addReproduciblePeakSet in ArchR, which generates pseudobulk replicates for user defined groups, calls peaks on these pseudobulk replicates using Macs2 to define a reproducible peak set for each group, and then merges the resulting peak sets for each group into a union peak set of fixed width peaks. For this peak calling step, cells were divided into groups based on T-cell subtype. After calling Macs2, a peak matrix was constructed in ArchR using addPeakMatrix. Annotations of motifs present in the peak set were identified with addMotifAnnotations and the cisbp motif set. Then background peaks were identified with addBgdPeaks and ChromVar deviations were added with addDeviationsMatrix. Imputation weights for imputed Chrom-Var z-scores were computed using addImputeWeights with k, the number of nearest neighbors for smoothing, set to 15, sample cells set to the number of total number of T-cells, and default values for all other parameters. Imputed deviation z-scores were then plotted on the T-cell UMAP. Differential peaks between different T-cell subtypes were computed with getMarkerFeatures using the Wilcoxon test and with bias set to c("TSSEnrichment", "log10(nFrags)"). Hypergeometric enrichment of motifs in markerPeaks were computed with peakAnnoEnrichment.

Single-cell ATAC-seq: Analysis of Stromal Compartment

To analyze the stromal cells present in our scATAC-seq dataset, we first performed dimensionality reduction and clustering. The iterative LSI dimensionality reduction was computed using addIterativeLSI with 3 iterations, clustering resolutions of 0.1 and 0.2 after the first and second iteration respectively, 20000 variable features, sampleCellsPre set to NULL, and using dimensions 1–30. Clusters were then determined with addClusters using the Seurat method, a resolution of 1.0, and nOutlier set to 50. As was done for the immune cells, we identified marker gene activity scores for each cluster and examined gene activity scores of known marker genes. For the stromal cells, one cluster was identified as a likely doublet cluster and removed. Dimensionality reduction and clustering was then repeated with identical parameters except a resolution of 1.1 was used in the final clustering step. A UMAP dimensionality reduction was then computed using addUMAP with nNeighbors of 35, minDist of 0.5, and the cosine metric.

To assign cell type annotations to the stromal clusters, gene activity scores for known marker genes were examined for different scATAC clusters. In the stromal annotation, we note that it is difficult to differentiate myofibroblasts from smooth muscle cells in single-cell data and while we labeled the corresponding clusters as myofibroblasts here, we cannot exclude one or more of these clusters being smooth muscle cells. The scATAC data was also integrated with snRNA from this study using the ArchR function addGeneIntegrationMatrix, enabling labeling of the scATAC cells based on the nearest snRNA cells. Similar to the immune cells, marker gene activity scores were used for initial annotation while labels from the RNA integration were used to validate and refine the initial annotations to produce the final cell type annotations.

Following cell type annotation, a stromal cell peak set was generated by first running addGroupCoverages and grouping by CellType and then running addReproduciblePeakSet. The getGroupSE function with divideN = TRUE was used to get a normalized aggregate peak by counts matrix for different cell types. Pearson correlations between accessibility in different cell types was computed with the cor function in R. To identify markerPeaks for the stromal compartment, getMarkerFeatures was run using the Wilcoxon test method and with TSSEnrichment and log10(nFrags) provided as bias parameters. Marker peaks with FDR <= 0.1 & $\log_2$FC >= 0.5 were visualized on the heatmap in Figure 2. Motif annotations using cisbp motifs were then added to the project and enrichment of motifs in marker peaks for each cell type were identified with the ArchR function enrichMotifs and cutoffs of FDR <= 0.1 & $\log_2$FC >= 0.5. ChromVAR deviations were then computed using addBgdPeaks and addDeviationsMatrix. Putative peak-to-gene links were identified with the ArchR function addPeak2GeneLinks with default parameters. While the above analyses highlight TF motifs that are associated with chromatin accessibility in different populations of stromal cells, many TFs share similar motifs, so identification of the precise factors that are functional in these cell types is aided by also examining TF expression. Thus, TFs likely regulating chromatin accessibility were determined exactly following the ArchR manual for identifying TF Regulators.

Single-cell ATAC-seq: Stromal Trajectory Analysis

The stromal cell trajectory from villus fibroblasts to CAFs was constructed by defining the trajectory as trajectory <- c("Villus Fibroblasts WNT5B+", "Inflammatory Fibroblasts", "Cancer Associated Fibroblasts") and then running the ArchR function addTrajectory. Trajectory heatmaps for the peakMatrix, motifMatrix, and GeneIntegrationMatrix were then plotted using getTrajectory and plotTrajectoryHeatmap. For this analysis, cells are grouped into high granularity groupings (100 groups along a trajectory of 3381 cells) and features are smoothed across a rolling average of 9 bins.

Single-cell ATAC-seq: Peak calling for epithelial compartment

To generate a union peak set representing all samples and cell types present in the epithelial compartment, we wanted to ensure that we captured sample- and cell-type-specific peaks. To accomplish this, we divided the cells into groups, generated pseudobulk replicates for each group, called peaks on the pseudobulk replicates, generated a reproducible peak set for each group using the peaks called for the pseduobulk replicates, and then iteratively merged the peaks sets for each group into a union peak set using the approach previously described for scATAC data and implemented in ArchR [20,21]. When selecting groups of cells for peak calling we choose not to create distinct groups for each epithelial cell type from each scATAC experiment because (A) this would result in a total of 979 groups (89 * 11 cell types) and (B) some of these groups would have very few cells and thus insufficient coverage to call peaks. To circumvent this, we opted for a mix of sample specific and cell type specific groupings. The following cell types consisted of a larger number of cells, so were initially divided into groups of each cell type from each sample: Stem, TA2, TA1, Enterocyte Progenitors, Immature Enterocytes, Enterocytes, Secretory TA, Immature Goblet, Goblet. The remaining cell types were divided into groups based on whether they originated from Normal, Unaffected, Polyp, or CRC samples (as defined by gross phenotype). After this initial classification, groups with fewer than 300 cells were identified. To preserve sample specific information, we first merged groups with their nearest cell type in the normal differentiation trajectory (e.g., if there were <300 enterocytes from a sample, that group was combined with the immature enterocyte group from the same sample). This was done iteratively with the following regrouping rules Enterocytes > Immature Enterocytes, Enterocyte Progenitors > Immature Enterocytes, TA1 > TA2, TA2 > Stem, Stem > TA2, Secretory TA > Immature Goblet, Goblet > Immature Goblet for groups with less than 300 cells prior to this step. For the cell type disease state groupings, there were insufficient cells for some of the enteroendocrine and Best4+ enterocyte groups, so normal and unaffected enteroendocrine groups were combined, polyp and CRC enteroendocrine groups were combined, and polyp and CRC Best4+ enterocyte groups were combined. After this step, we again identified any groups that did not have at least 300 remaining cells, and then combined the groups as follows:

1) Patient F Immature Enterocytes groups with fewer than 300 cells were merged into a single group.
2) Patient A002 Immature Enterocytes groups with fewer than 300 cells were merged into a single group.
3) Patient A014 Immature Enterocytes groups with fewer than 300 cells were merged into a single group.

4) Immature Goblet groups from A002 Unaffected samples with fewer than 300 cells were merged into a single group.
5) CRC-1-8810-Immature Goblet, CRC-2-15564-Immature Goblet, CRC-3-11773-Immature Goblet, and A001-C-007-Immature Goblet were merged together.
6) F091-Immature Goblet and F034-Immature Goblet groups were merged together.
7) F034-TA2 and F091-TA2 groups were merged together.
8) A015-C-001-Immature Goblet and A015-C-002-Immature Goblet groups were merged together.
9) A015-C-001-TA2 and A015-C-002-TA2 groups were merged together.
10) A014-C-008-Immature Goblet and A014-C-108-Immature Goblet groups were merged together.
11) A002-C-021-Immature Goblet and A002-C-016-Immature Goblet groups were merged together.

This process resulted in a total of 271 cell groupings with an average 1,327 cells per grouping. After defining these groupings, the ArchR functions addGroupCoverages followed by addReproduciblePeakSet were run using these groupings to group the cells for peak calling and setting all other parameters to their default values.

Single-cell ATAC-seq: Identification of Differential Peaks

To compute pairwise differential tests, the ArchR function getMarkerFeatures was used with testMethod set to Wilcoxon and bias set to c("TSSEnrichment", "log10(nFrags)") and useGroups and bgdGroups set to be the two samples being tested.

Single-nuclei RNA-seq: Dimensionality Reduction, Clustering, and Annotation of Immune and Stromal Compartments

Cells from the immune and stromal subcompartments were analyzed with Seurat's standard analysis pipeline. First, data was normalized with NormalizeData and scaled with ScaleData. Principal components were then computed on the scaled data and a UMAP was generated from the resulting PCs. Clusters were identified with the surat function FindNeigbors and FindClusters with a resolution of 0.5 for the immune cells and 0.5 for the stromal cells. Markers for each cluster were identified with the Seurat function FindMarkers. Low quality or likely doublet clusters were identified based on no expression of any marker genes or expression of marker genes from multiple cells types, as discussed below.

After removal of low quality/doublet cluster, the dimensionality reduction and clustering was repeated on the resulting cells as described above but with a final clustering resolution of 2.1 for the immune cells and 1.0 for the stromal cells. Following clustering, immune and stromal clusters were annotated based on expression of known marker genes in each cluster. In the stromal annotation, we note that it is difficult to differentiate myofibroblasts from smooth muscle cells in single-cell data and while we labeled the corresponding clusters as myofibroblasts here, we cannot exclude one or more of these clusters being smooth muscle cells. To support the immune annotations, the snRNA immune dataset was also annotated using SingleR (version 1.4.1)[22] in R version 4.0.2. The HumanPrimaryCellAtlasData was used as a reference and was subsetted to include only reference data with the following main labels: "DC", "Epithelial_cells", "B_cell",

"Neutrophils", "T_cells", "Monocyte", "Endothelial_cells", "Neurons", "Macrophage", "NK_cell", "BM", "Platelets", "Fibroblasts", "Astrocyte", "Myelocyte", "Pre-B_cell_CD34-", "Pro-B_cell_CD34+", and "Pro-Myelocyte." The cell type of each snRNA immune cell was then predicted with the SingleR function and labels set to label.fine. The most common cell types predicted by singleR in each cluster generally agreed with our manual annotation (Figure S1E). Following annotation, markers for each cell type were identified with the seurat function FindAllMarkers.

Single-nuclei RNA-seq: Data Visualization
All Dot Plots were generated using the Seurat function DotPlot and all plots of expression on the UMAP projection were generated with the Seurat function FeaturePlot.

Single-nuclei RNA-seq: GO term enrichment
Following identification of clusters of genes that are differential along the continuum, we tested if any gene ontology (GO) terms were enriched in these clusters of genes. To the list of genes in each cluster was provided to the goana function in limma[23]. We retained all biological process GO terms with at least 3 and less than or equal to 200 genes, and plotted the GO terms with the most significant p-values in any clusters as determined by goana.

Single-nuclei RNA and ATAC: Removal of possible doublet cells
Following initial clustering of the data into stromal, immune, and epithelial subtypes, we examined the data for any low quality or likely doublet clusters. This involved first identifying marker genes for the snRNA data and marker gene scores for the scATAC data (FDR <= 0.01 & $\log_2$FC >= 1; Wilcoxon test). We then compared the marker genes or marker gene scores to genes that were previously found to be specific to colon cell types in a different scRNA-seq dataset (Smillie et. al.). In cases where clusters either did not have any expected differential marker genes (e.g. an immune cell cluster with no significant immune cell markers) or had differential markers that were specific to a cell type from a different compartment (e.g. epithelial marker genes in a immune cluster), those clusters were thought to be low-quality or doublet clusters and removed prior to downstream analysis.

This process resulted in removal of a few small clusters from each compartment. For the stromal RNA data, clusters that originally clustered with the stromal RNA data, but when compared to other stromal cells had much higher expression of epithelial marker genes, leading us to believe that they likely represent doublets that are a mix of the stromal and epithelial lineages. For the stromal ATAC data, 1 cluster that initially clustered with the stromal cells was removed. Following initial dimensionality reduction and clustering this cluster had significantly higher gene scores for a number of genes that are expected to be specifically expressed on immune cells, including CD3D, CD3E, and CD53. For the immune scATAC data, three clusters that originally clustered with the immune cells were removed from the scATAC data. These clusters had very few immune specific marker genes. One of the clusters had significantly differential gene scores for TNS1 which was previously found to be a marker for pericytes, but no significantly differential gene scores for any previously defined immune specific genes. A second cluster had significantly differential gene scores for a number of non-immune genes including PROX1 (Best4+

enterocytes), FAM183A (secretory epithelial cells), SFTA1P (fibroblasts), and TFPI2 (fibroblasts), which would be unexpected for a population of immune cells. For immune RNA data, 4 small clusters were removed. Marker genes for these clusters suggested they are unlikely to be pure immune populations and included the epithelial genes EPCAM, ELF3, KLF5 (first removed cluster), PIGR, ELF3, KLF5 (cluster 2), and LGALS4, KRT19, CA2 (cluster 3) and the myofibroblast genes ACTA2 and MYH11 (cluster 4).

Single-nuclei RNA and ATAC: Discussion of possible technical issues
All single-cell studies are at risk of batch effects or technical factors contributing signals to the data that may alter the findings. Here we discuss some possible technical factors and evidence we have that they are not significant confounding factors.

Batch effects: One advantage of this work is that we have matched unaffected tissues and polyps for many of the patients. For these cases, unaffected tissues tend to look more like unaffected tissues from other patients than like polyps from the same patient suggesting that patient identity is a small batch effect relative to the biological differences between disease states. For example, the cell type abundances from unaffected polyps demonstrate clear trends across patients that are distinct from those observed in polyps (Figure S2). Specifically, we observe many more B cells in unaffected tissues, which is consistent across patients, than we see in polyps. Within the stromal compartment, we observe populations of fibroblast subtypes that are greatly represented across unaffected tissues (e.g crypt fibroblasts 4 in yellow), but have generally much lower representation across all polyps.
Additionally, we observed that, in general, clustering is not driven by sample or donor, but by disease state, with the exception of the epithelial cells from some cancers largely clustering on their own (Figure S3I).
We also tested batch correction methods to see if they would alter clustering of the cells, and found that overall they had relatively little effect on the clustering of the data. For example, when applying Harmony batch correction to the stromal data, the clustering of the cells is largely unchanged, and we do not observe clusters of different cell types merging together (Figure S1M).

Mitochondrial content: Regarding mitochondrial composition, we found that levels of mitochondrial RNA were typically low and did not drive clustering. For most samples the median percent of total reads that were mitochondrial reads per cell was less than 1 percent, and even the sample with the most mitochondrial content had a mean of just over 2 percent (Figure S1B).

Stress Response in Clinical Samples: Interferon gamma pathways can be activated in clinical samples. To examine if samples from any disease state have higher expression of interferon gamma pathways, we plotted module scores, computed with AddModuleScore, for two two interferon gamma gene sets, ST_INTERFERON_GAMMA_PATHWAY and REACTOME_INTERFERON_GAMMA_SIGNALING, for immune cells from normal, unaffected, polyp, and CRC. We did not observe substantial differences in interferon gamma pathways based on disease state, and observed slightly higher typeII interferon pathway activity in dendritic cells when comparing activity between cell types (Figures S1N and S1O).

Single-nuclei RNA and ATAC: Differential Cell Type Abundance Testing

Differential cell type abundance testing was carried out in two ways. The first was Wilcoxon testing on the fraction of each cell type in each compartment with Bonferroni corrections included for multiple tests of the same cell type. Replicates were merged prior to Wilcoxon testing and Wilcoxon testing was only performed on samples with at least 50 cells in a given compartment. Two-sided Wilcoxon testing was performed with the function compare_means in the ggpubr package with method = 'wilcox.text' and p.adjust.method = "bonferroni". Wilcoxon testing is a straightforward approach that is frequently applied in determining differences in cell type abundance; however, it has limitations including not accounting for possible batch effects and the dependence of the method on cluster assignments. To account for these limitations and provide additional evidence for changes in cell type abundance, we applied Milo, a method that fits a generalized-linear model (GLM) to determine differential abundance of cell neighborhoods on a k-nearest neighbor graph while correcting for processing batch (Figure S4B) [24]. When running Milo, we first created a SingleCellExperiment object containing the reduced LSI dimensions for the scATAC data. We created a Milo object with the function Milo and then built the graph with the function buildGraph with k=100, d=30, and reduced.dim=LSI. makeNhoods was then run with prop=0.1, k=100, d=30, and reduced.dim=LSI. When running Milo to compare discrete disease states, testNhoods was run with design = ~ ATAC_Processing_Date + DiseaseState. When running Milo to determine changes along the continuum, testNhoods was run with design = ~ ATAC_Processing_Date + continuum with continuum being the x-value of the nearest point in the spline fit to the malignancy continuum. The results of these two methods were generally consistent. For example, neighborhoods of regulatory T-cells (Tregs) were enriched in polyps relative to unaffected tissues in the Milo analysis, and Tregs were significantly more abundant by Wilcoxon testing. Similarly, neighborhoods of naive B, memory B, and GC cells were enriched in unaffected tissues relative to polyps, and were also significantly enriched by Wilcoxon testing (Figures S4A and S4B).

Single-nuclei RNA and ATAC: Assessment of cellular coherence among epithelial cells

For the RNA data, we first defined a stem cell gene signature by identifying significantly differentially expressed genes in stem/stem-like cells relative to all other cell types using the Seurat function FindMarkers with test.use set to "MAST" and then selecting genes with logFC>=0.5 and padj<=0.05. We then assigned each single cell in the epithelial compartment a score based on their normalized average expression for each gene using the function AddModuleScore. An analogous procedure was performed for the scATAC data. We first computed marker peaks for stem/stem-like cells relative to all other cell types. We then computed the number of Tn5 insertions in significantly differential stem cell peaks for each cell in our dataset and normalized each cell by the total number of fragments. The range of ATAC and RNA "scores" were each divided into 50 bins and the fraction of cells falling into each bin for each sample was plotted. To order the samples, the percent of cells in each RNA or ATAC bin was multiplied by the bin number (1 being least stem-like and 50 being most stem-like) and these values were then summed to give a total for each sample, resulting in higher totals for samples with more cells in more stem-like bins. Only samples with at least 100 RNA and 100 ATAC cells are included.

We also performed the analysis with an alternative approach, where, for each sample, we place cells into bins based on the nearest normal cells when they are projected into the normal colon LSI subspace. We then order samples by computing the a product of a subset of the cell types and the percent of the cell type in each bin (3*stem + 2*cyclingTA + 1*TA2 - 2*enterocyte), which gives more weight to more immature cells and penalizes samples with more mature cells.

CODEX: Tissue prepping and CODEX multicycle imaging
Detailed stepwise Codex protocols including antibody conjugation and instrument setup for running imaging multicycles can be found online https://d1fgrgbaaj3jvk.cloudfront.net/wp-content/uploads/2020/04/CODEX-User-Manual-Rev-B.0.pdf. Briefly, tissue sections ~8 µm thick were cut from Optimal Cutting Temperature blocks (Tissue-Tek® O.C.T., Sakura Finetec, USA) using a cryostat (Leica CM1950, Leica Biosystems, USA), and mounted on 22 mm X 22 mm poly-L-lysine coated coverslips (Electron Microscopy Sciences, PA, USA). The sample coverslips were cryostored at -80 °C, until needed. Sample coverslips were obtained from the freezer and placed on 1-2 cm Drierite beads for 2 mins, followed by 10 mins incubation in acetone. The acetone was dried by placing the coverslips facing up in a humidity chamber for 2 mins. The tissue sections were incubated in 5 ml Hydration buffer for 2 mins (twice) and fixed using 1.6% Paraformaldehyde (PFA) for 10 mins, after which the fixative was thoroughly removed by rinsing in the same Hydration buffer. The coverslips were incubated in 5 ml Staining buffer for 20 mins. In the meantime, a stock solution of Codex blocking buffer was prepared by mixing 362 µl of Staining buffer and 9.5 µl each of N, G, J, S blockers (per 2 samples). The volume of antibody per sample coverslip was calculated and subtracted from the Codex blocking buffer. An antibody cocktail solution was prepared by pipetting each antibody into the Codex blocking buffer. In this case, no custom conjugation was necessary. Several barcoded antibodies including CD3-BX015 (UCHT1)—Cy5-RX015 and PD-1/CD279-BX014 (EH12.2H7)—Atto 550-RX014 were purchased from Akoya Biosciences. Both antibodies were diluted to 1:200. The sample coverslips were placed in the humidity chamber and the antibody cocktail staining solution (200 µl per tissue sample) was dispensed on the coverslip and incubated for 3 hours at room temperature. Subsequently, unbound antibodies were removed by rinsing the coverslips in 5 ml Staining buffer for 2 mins (twice). The samples were placed in post-staining fixative solution (1 ml of 16% PFA + 9 ml of Storage buffer) for 10 mins and washed thoroughly with 1x Phosphate buffer saline (PBS) to remove the fixative. Subsequently, the tissue sections were fixed with ice cold methanol for 5 mins at 4 °C and washed with 1x PBS. The samples were incubated in a final fixative reagent (20 µl of Codex fixative reagent + 1 ml of 1x PBS) for 20 mins, washed thoroughly with 1x PBS before placing in the storage buffer (up to 2 weeks at 4 °C). A Codex microfluidic instrument (Akoya Biosciences Inc., CA, USA) integrated with an inverted fluorescence microscope through a custom stage insert was used to automate Codex buffer exchange and image acquisition. The tissue sections (on coverslip) were placed into the stage insert and imaged over 4 cycles of Codex protocol. The first and the last cycles were basically blank cycles and also used for image registration and image alignment. A Keyence BZ-X810 fluorescent microscope configured with 4 fluorescent channels (DAPI, FITC, Cy3, Cy5) and equipped with Nikon objective lens (20x) – CFI Plan Apo 20x/0.75NA was used to collect images. The exposure time for CD3-BX015 (UCHT1)—Cy5-RX015 and PD-1/CD279-BX014 (EH12.2H7)—Atto 550-RX014 were 450 ms and 500 ms respectively. Tissue sections were imaged in a 5x7 tiled acquisition at 377 nm/pixel resolution

and 9 z-planes per tile. The tile overlap was preset at 30%. The image data and experiment.json files were transferred to Codex Analysis Manager, (CAM, Akoya Biosciences Inc.) for further processing of the raw images, which were then subjected to background subtraction, shading correction, along with deconvolution to remove out-of-focus light using a Microvolution software available from http://www.microvolution.com/. After drift-compensation and stitching, the best focal plane of vertical image stacks collected at each acquisition were chosen for cell segmentation using a gradient-tracing watershed algorithm on the nuclear staining (radius set to 6 pixels) as described in Golstev et al., 2019.

CODEX: Reporter plate for corresponding antibodies
A stock solution to run 4 cycles was prepared by adding 1220 µl of nuclease-free water, 150 µl of 10x Codex buffer, 125 µl of Assay reagent, and 4 µl of nuclear stain. Two forty microliters of the master mix were dispensed into individual wells followed by 5 µl of the corresponding reporter dyes. The reporter solutions were mixed and pipetted into individual wells of a 96-well plate, covered with a foil seal, and stored at 4 °C for imaging.

Whole Genome Sequencing: Sample Processing and Library Preparation
Patient tissue was either removed from the colon with a surgical scalpel blade or blood was drawn using purple top tubes. Immediately, the solid tissues were placed into cryovial tubes and quickly into a portable liquid nitrogen tank. After the purple top tubes were centrifuged for 10 minutes at 7C at 1200g, the top layer of plasma was removed and the central layer of buffy coat was preserved in cryovials on dry ice. We created either fine powders or small chunks of the flash frozen tissue using a ceramic mortar and pestle and liquid nitrogen as input for DNA extraction with the Qiagen All-prep kits (cat. 80204). Buffy coat DNA was isolated by thawing the sample then placing 200 ul directly into 600 µl Buffer RLT with β-ME at 1:100 dilution followed by the All-prep kit instructions.

The DNA was sequenced on the Illumina NovaSeq 6000 after library preparation using either the NEBNext DNA Library Prep Kit (cat. no. E7645S) or TruSeq Nano DNA Kit (cat. No. 20015964) following the manufacturers guidelines. The DNA extracted from FFPE was first treated with S1 Nuclease from Thermo Scientific (cat. no. EN0321) followed by NEBNext FFPE DNA Repair Mix (cat. no. M6630) then NEBNext DNA Library Prep Kit.

Whole Genome Sequencing: Somatic Mutation Detection
SNVs and indels were detected using GATK v.4 Mutect2 in multi-sample mode per patient in which "-normal" was the .bam file from the buffy coat from that patient. The --germline-resource was af-only-gnomad.hg38.vcf. To remove FFPE artifacts, we ran Mutect2 with the argument --f1r2-tar-gz then used LearnReadOrientationModel and MergeMutectStats. Filtering mutations was done using FilterMutectCalls with the following arguments: -stats ${*patient ID*}.merged.stats , --orientation-bias- artifact-priors ${*patient ID*}-read-orientation-model.tar.gz , --max-events-in-region 30, --max-alt-allele- count 2, -XL hg38-blocklist.v2.bed , -XL ENCFF356LFX.bed. The resulting mutations were left-aligned and multi-allelic mutations were separated with LeftAlignAndTrimVariants. We annotated the mutations with Annovar using the hg38 reference: refGene, clinvar_20170905, cosmic70, dbnsfp33a, cytoBand, avsnp150. Any mutations found in

the patient buffy coat samples were also removed from any downstream steps. Only mutations with >=3 variant-supporting reads (altc) and >=10 total supporting reads were considered "present". Copy number variants (CNVs) and estimates for purity and ploidy were derived using Titan.

REFERENCES

18. Corces, M. R. *et al.* The chromatin accessibility landscape of primary human cancers. *Science* **362**, (2018).
19. Yost, K. E. *et al.* Clonal replacement of tumor-specific T cells following PD-1 blockade. *Nat. Med.* **25**, 1251–1259 (2019).
20. Granja, J. M. *et al.* Single-cell multiomic analysis identifies regulatory programs in mixed-phenotype acute leukemia. *Nat. Biotechnol.* **37**, 1458–1465 (2019).
21. Granja, J. M. *et al.* ArchR is a scalable software package for integrative single-cell chromatin accessibility analysis. *Nat. Genet.* **53**, 403–411 (2021).
22. Aran, D. *et al.* Reference-based analysis of lung single-cell sequencing reveals a transitional profibrotic macrophage. *Nat. Immunol.* **20**, 163–172 (2019).
23. Ritchie, M. E. *et al.* limma powers differential expression analyses for RNA-sequencing and microarray studies. *Nucleic Acids Res.* **43**, e47 (2015).
24. Dann, E., Henderson, N. C., Teichmann, S. A., Morgan, M. D. & Marioni, J. C. Differential abundance testing on single-cell data using *k*-nearest neighbor graphs. *Nat. Biotechnol.* **40**, 245–253 (2022).
