# Supplementary material for: Single-cell analyses define a continuum of cell state and composition changes in the malignant transformation of polyps to colorectal cancer
Source: Nat Genet. 2022 Jun 20;54(7):985–95. doi: 10.1038/s41588-022-01088-x (PMC9279149; doi:10.1038/s41588-022-01088-x)
Supplement: Supplementary file 2 — Reporting Summary [file 41588_2022_1088_MOESM2_ESM.pdf]

nature research

Corresponding author(s): William Greenleaf, Michael Snyder

Last updated by author(s): Apr 25, 2022

Reporting Summary

Nature Research wishes to improve the reproducibility of the work that we publish. This form provides structure for consistency and transparency in reporting. For further information on Nature Research policies, see our Editorial Policies and the Editorial Policy Checklist.

Statistics

For all statistical analyses, confirm that the following items are present in the figure legend, table legend, main text, or Methods section.

n/a	Confirmed	
☐	☒	The exact sample size (*n*) for each experimental group/condition, given as a discrete number and unit of measurement
☐	☒	A statement on whether measurements were taken from distinct samples or whether the same sample was measured repeatedly
☐	☒	The statistical test(s) used AND whether they are one- or two-sided<br>*Only common tests should be described solely by name; describe more complex techniques in the Methods section.*
☒	☐	A description of all covariates tested
☐	☒	A description of any assumptions or corrections, such as tests of normality and adjustment for multiple comparisons
☐	☒	A full description of the statistical parameters including central tendency (e.g. means) or other basic estimates (e.g. regression coefficient) AND variation (e.g. standard deviation) or associated estimates of uncertainty (e.g. confidence intervals)
☐	☒	For null hypothesis testing, the test statistic (e.g. *F*, *t*, *r*) with confidence intervals, effect sizes, degrees of freedom and *P* value noted<br>*Give P values as exact values whenever suitable.*
☒	☐	For Bayesian analysis, information on the choice of priors and Markov chain Monte Carlo settings
☒	☐	For hierarchical and complex designs, identification of the appropriate level for tests and full reporting of outcomes
☐	☒	Estimates of effect sizes (e.g. Cohen's *d*, Pearson's *r*), indicating how they were calculated

Our web collection on statistics for biologists contains articles on many of the points above.

Software and code

Policy information about availability of computer code

Data collection	Code for generating fragments files for scATAC and counts matricies for single cell RNA was obtained from 10x genomics (go.10xgenomics.com/scATAC/cell-ranger-ATAC and https://support.10xgenomics.com/single-cell-gene-expression/software/pipelines/latest/what-is-cell-ranger).
Data analysis	cellranger-atac-1.2.0 – alignment of ATAC data and generation of fragments files<br>cellranger-3.1.0 – alignment of RNA data and generation of counts matrices<br>macs2 2.1.1.20160309 – Software for peak calling<br>R version 3.6.1 – R environment for all custom code except running singleR.<br>R version 4.0.2 – R environment for running singleR.<br>ArchR - 0.9.5 - Software for analysis of scATAC-seq data.<br>Seurat_3.1.1 – Software for analysis of scRNA-seq data.<br>DoubletFinder_2.0.3 – Software for doublet removal for scRNA-seq<br>BSgenome.Hsapiens.UCSC.hg38_1.4.1 – Package containing genomic DNA sequences<br>TCGAbiolinks_2.12.6 – Software for analysis of DNA methylation data.<br>limma_3.40.6 – Software used for GO enrichments<br>Rcpp_1.0.4.6 – Software for C++ in R<br>SingleR_1.4.1 – Software for automated cell annotation<br>edgeR_3.26.8 – Used for analysis of single-cell data.<br>harmony_1.0 – Software used for batch correction.<br>Mutect2 (GATK v4) – Used for analysis of whole genome data.<br>Custom code for generating the malignancy continuum is available on GitHub (https://github.com/winstonbecker/scCRC_continuum).

For manuscripts utilizing custom algorithms or software that are central to the research but not yet described in published literature, software must be made available to editors and reviewers. We strongly encourage code deposition in a community repository (e.g. GitHub). See the Nature Research guidelines for submitting code & software for further information.

Data

Policy information about availability of data

All manuscripts must include a data availability statement. This statement should provide the following information, where applicable:

- Accession codes, unique identifiers, or web links for publicly available datasets
- A list of figures that have associated raw data
- A description of any restrictions on data availability

Sequencing data has been deposited in the Gene Expression Omnibus (GEO) with the accession code GSE201349. Original data generated in this study are also available on the Human Tumor Atlas Network (HTAN) Data Portal (unaffected FAP tissues, polyps, and CRCs; https://data.humantumoratlas.org/ under the HTAN Stanford Atlas) and the HuBMAP data portal (normal colon tissues; https://portal.hubmapconsortium.org/ under the Stanford TMC). Unique IDs for accessing the HTAN datasets are listed in Supplementary Table 3 and unique IDs for accessing the HuBMAP datasets are listed in Supplementary Tables 4 and 5. Receptor ligand pains from the Fantom5 database were downloaded from https://fantom.gsc.riken.jp/5/suppl/Ramilowski_et_al_2015/. Clustered TF motifs can be downloaded from https://www.vierstra.org/resources/motif_clustering#downloads. Seurat objects for previously published single-cell colon data were downloaded from https://github.com/cssmillie/ulcerative_colitis. Counts matrices and T-cell annotations for cells from BCC are available on GEO with accession number GSE12381315. TCGA DNA methylation data can be downloaded from the GDC data portal (https://portal.gdc.cancer.gov/).

Field-specific reporting

Please select the one below that is the best fit for your research. If you are not sure, read the appropriate sections before making your selection.

☒ Life sciences ☐ Behavioural & social sciences ☐ Ecological, evolutionary & environmental sciences

For a reference copy of the document with all sections, see nature.com/documents/nr-reporting-summary-flat.pdf

Life sciences study design

All studies must disclose on these points even when the disclosure is negative.

Sample size	Sample size was set based on the availability of polyps collected. We aimed to maximize the number of polyps assayed, as we were primarily interested in identifying features of the precancerous state. A smaller number of normal tissues and CRCs were collected to facilitate analysis of the polyps and to help define the precancerous continuum. The sample size in this study was sufficient to define a continuum from normal to cancer and to identify significant changes in composition between disease states.
Data exclusions	All datasets generated that did not fail experimentally (e.g. overloaded sample) were included in the study.
Replication	Replicate single cell ATAC datasets were generated for 4 samples and produced highly concordant results. Replicates for additional single-cell experiments were not performed as technical replicates are less informative than additional samples with different disease states. Further, using multimodal data (scATAC and snRNA) allows us to highlight results that are concordant between the two methods. Selected findings (e.g. PD1 expression) were also validated with orthogonal assays.
Randomization	There was no randomization into experimental groups as experiments on all samples in this study were performed the same way.
Blinding	No blinding was performed in this study that focused on deep characterization of polyps, CRCs, and normal tissues at a single point in time. No differential clinical intervention was performed or was being compared in this study.

Reporting for specific materials, systems and methods

We require information from authors about some types of materials, experimental systems and methods used in many studies. Here, indicate whether each material, system or method listed is relevant to your study. If you are not sure if a list item applies to your research, read the appropriate section before selecting a response.

Materials & experimental systems

n/a	Involved in the study	
☐	☒	Antibodies
☒	☐	Eukaryotic cell lines
☒	☐	Palaeontology and archaeology
☒	☐	Animals and other organisms
☐	☒	Human research participants
☒	☐	Clinical data
☒	☐	Dual use research of concern

Methods

n/a	Involved in the study	
☒	☐	ChIP-seq
☒	☐	Flow cytometry
☒	☐	MRI-based neuroimaging

Antibodies

Antibodies used	CD3-BX015 (UCHT1)—Cy5-RX015; Supplier: Akoya Biosciences, Clone: UCHT1; Catalog number: 4350008<br>PD-1/CD279-BX014 (EH12.2H7)—Atto 550-RX014; Supplier: Akoya Biosciences, Clone: EH12.2H7; Catalog number: 4250010
Validation	The CD3 and PD-1 antibodies were pre-validated and conjugated by Akoya Biosciences, which were purchased for running the CODEX experiments.

Human research participants

Policy information about studies involving human research participants

Population characteristics	Patients in this study include patients with FAP seen at Stanford healthcare, 1 patient without FAP undergoing routine screening colonoscopy at Stanford healthcare, patients with CRC with samples deposited in the Stanford Tumor bank, and patients without FAP or colon cancer at WUSTL. Ages of patients included ranged from 20–78. The study included 9 male and 6 female patients. 6 patients were White, 5 were Hispanic/Latino, 1 White/Asian, 1 Black, and 1 Other. Please see the supplemental table for additional information.
Recruitment	For the FAP patients, given that this is a relatively rare condition, no criteria other than FAP were required for recruitment. Eligible patients undergoing colonoscopy, pouchoscopy, or colectomy were identified in the Stanford Cancer Genetics clinic, the Stanford Gastroenterology and Hepatology service, or the Stanford Adult and Pediatric Surgery service. Eligible patients were notified of their eligibility to participate in research. Over the phone, the description, risks, benefits, and alternatives of participating in the research study were described and a copy of the full consent form was sent to them via email. On they day of the procedure, they met with a clinical research coordinator to answer questions and sign the consent. This recruitment strategy only includes patient's seen at Stanford healthcare, which is one potential source of selection bias. For polyps from non-FAP patients, they were obtained from patients undergoing screening colonoscopy at Stanford with no attempt to exclude donors on the basis of age, gender, or sex. Healthy controls were recruited at WUSTL, and no attempt was made to exclude healthy donors on the basis of age, gender, or sex. A subset of CRC samples were obtianed from the Stanford tumor bank, again with no attempt to exclude patients on the basis of age, gender, or sex.
Ethics oversight	The study was approved by the Stanford IRB and informed consent was obtained from all patients.

Note that full information on the approval of the study protocol must also be provided in the manuscript.
